# Investigating Generalized Performance of Data-Constrained Supervised Machine Learning Models on Novel, Related Samples in Intrusion Detection

**DOI:** 10.3390/s23041846

**Published:** 2023-02-07

**Authors:** Laurens D’hooge, Miel Verkerken, Tim Wauters, Filip De Turck, Bruno Volckaert

**Affiliations:** IDLab, Department of Information Technology, Ghent University-imec, 9052 Gent, Belgium

**Keywords:** CIC-IDS2017, CSE-CIC-IDS2018, CIC-DoS2017, CIC-DDoS2019, cybersecurity, generalization, intrusion detection, network security, network traffic classification, supervised machine learning

## Abstract

Recently proposed methods in intrusion detection are iterating on machine learning methods as a potential solution. These novel methods are validated on one or more datasets from a sparse collection of academic intrusion detection datasets. Their recognition as improvements to the state-of-the-art is largely dependent on whether they can demonstrate a reliable increase in classification metrics compared to similar works validated on the same datasets. Whether these increases are meaningful outside of the training/testing datasets is rarely asked and never investigated. This work aims to demonstrate that strong general performance does not typically follow from strong classification on the current intrusion detection datasets. Binary classification models from a range of algorithmic families are trained on the attack classes of CSE-CIC-IDS2018, a state-of-the-art intrusion detection dataset. After establishing baselines for each class at various points of data access, the same trained models are tasked with classifying samples from the corresponding attack classes in CIC-IDS2017, CIC-DoS2017 and CIC-DDoS2019. Contrary to what the baseline results would suggest, the models have rarely learned a generally applicable representation of their attack class. Stability and predictability of generalized model performance are central issues for all methods on all attack classes. Focusing only on the three best-in-class models in terms of interdataset generalization, reveals that for network-centric attack classes (brute force, denial of service and distributed denial of service), general representations can be learned with flat losses in classification performance (precision and recall) below 5%. Other attack classes vary in generalized performance from stark losses in recall (−35%) with intact precision (98+%) for botnets to total degradation of precision and moderate recall loss for Web attack and infiltration models. The core conclusion of this article is a warning to researchers in the field. Expecting results of proposed methods on the test sets of state-of-the-art intrusion detection datasets to translate to generalized performance is likely a serious overestimation. Four proposals to reduce this overestimation are set out as future work directions.

## 1. Introduction

Every day, a larger and more varied collection of devices is connected to the Internet. This connectivity exposes the same devices to the malicious actors who are active on the Internet, continually looking for exploitable targets. The discovery, classification and tracking of malicious activity against any host or network thus becomes increasingly important. Creating this type of defense has been an active research field since at least 1985 [1,2]. At the time, connectivity was not widespread, so researchers formulated their defense in terms of monitoring the devices for intrusions instead of the network. This type of defense is called host intrusion detection (HIDS), and its monitoring perimeter does not extend beyond the device itself. Common techniques include analysis of the operating system and the running programs in terms of system call usage, input/output behavior, resource utilization and system/application logs.

Because network connectivity kept increasing, a second branch that places communication between devices at its center was added. Network intrusion detection systems (NIDS) aim to model attacks from the information present at various abstraction levels of network traffic. At the highest level of resolution, deep packet inspection decides whether traffic is malicious or not based on the information encapsulated in the data portion of the network packets. This type of IDS is becoming more obsolete because many connections use encryption at least on the payload [3]. Packet-level IDS increases its view by including the protocol headers and other metrics to make a decision. Flow-level IDS no longer decides on the individual packets of a connection but judges them together as a flow record. Further abstractions are possible but uncommon.

At each resolution, a subdivision can be made into roughly two categories. Either the system operates based on a set of rules or it has a more abstract representation of legitimate behavior and reports the anomalous events. Traditionally, rule-based systems rely on a signature database with condensed, unique representations of known attacks. Packets or flows are matched against the signatures and rejected if they match an attack signature. Precision is the main advantage of this approach. The signature’s metadata immediately conveys the attack class, the specific attack and potentially even which strain of the attack. The main downside is the fragility of these systems. Attackers try to fool rule-based IDS by applying the smallest possible alteration that leads to a mismatch in the signature engine [4,5]. Defenders try to update or generalize the rules to be more robust against these attacks [6]. Both sides use more and more sophisticated (and computationally demanding) techniques to evade or capture one another. Both open-source and proprietary rule-based IDSs exist and they are commonly used as a layer of defense in current networks.

An IDS that relies on anomaly detection tries to model normality and reports the anomalous events. Under modern conditions this definition only includes unsupervised learning methods, which is overly restrictive. This is a historical artifact because early anomaly detection IDSs relied on thresholds (static at first, later dynamic) derived from the networks on which they operated. Many recent methods are based on supervised machine learning algorithms (ML), and the authors still classify their proposals within the anomaly detection IDS category. ML-based IDS promises to deliver models that have solid, general representations of just benign traffic (pure anomaly detection) or of benign and malicious traffic (supervised classification). If successful, ML-based IDSs would overcome the core deficiency of rule-based IDS and supersede them.

Unfortunately, ML-based IDS has been studied for well over twenty years, and adoption into open-source or proprietary network defense software is almost nonexistent. We posit that this is in part due to the way in which ML-based IDSs are evaluated and adopted by the research community. Well-adopted literature [7,8,9,10,11,12,13,14] from the state-of-the-art proposes novel (combinations of) algorithms and validates its methods by showing increases in classification metrics on a handful of academic benchmark datasets. Consistent increases in classification performance are easily recognized as advances in the field. However, these works do not include any form of testing beyond the datasets on which their models have been trained, validated and tested.

### 1.1. Research Contribution

This work is the first, to the authors’ knowledge, large-scale investigation into the interdataset generalizability of ML methods in intrusion detection. A varied set of well-established ML methods which have been part of many published works is tested for their ability to use their learned representations to recognize new samples from the same attack classes without retraining.

Binary classification models have been trained for every attack class present in CSE-CIC-IDS2018 (brute force, DoS, DDoS, botnet, Web attack and infiltration). Previous work [15] has shown that the baseline classification (standard intradataset generalization) of the same models on these attack classes is excellent. As a bonus, the models have shown equally great resistance to deliberate attempts to make classification more difficult (both by restricting sample and feature access).

Those positive signals give the impression that the models are capable of learning a robust, general representation of their attack class, but this article demonstrates that claim is false. The general performance across datasets is erratic and the resistance to data reduction does not carry through even though the samples are closely related. Despite the unpredictability, a subset of models for the network-centric attack classes (brute force, DoS, DDoS and botnet to some extent) do maintain their discriminating power on the novel samples.

### 1.2. Paper Outline

The remainder of this article is split into 6 other sections. The related work in Section 1.3 familiarizes the reader with existing, but largely theoretical, critiques of intrusion detection from multiple angles. In Section 2, we discuss the included datasets and the training/evaluation procedures. Most of the body of this article is spent on Section 3 and Section 4. The results start off with the baseline results as a point of reference (Section 3.2). The other subsections detail how well these pretrained models generalize to the samples from the respective attack classes of CIC-IDS2017 (Section 3.3) with additional testing for the network-centric attack classes DoS and DDoS classes (represented by CIC-DoS2017 Section 3.4 and CIC-DDoS2019 Section 3.5). The results have intermediate conclusions to maintain oversight.

Because the result section is in-depth and verbose, the discussion (Section 4) takes a different approach. It centers around the interdataset generalized results by comparing the top 3 baseline models to the top 3 generalized models in a table per target dataset and attack class. Although this representation hides the stability issues, it does give an immediate overview of potential general performance on each attack class. This article ends with an overarching conclusion in Section 5, followed by routes for further investigation into improving IDS models for reliable use (Section 5.1).

### 1.3. Related Work

The related work demonstrates the need for research into generalization from different perspectives. It starts by noting the attention that is given to robust, generalized performance in the computer vision (CV) and natural language processing (NLP) domains. Subsequently, the lack of experimental research into generalization in network intrusion detection is highlighted with some examples.

#### 1.3.1. Generalization Research in Prominent ML Domains

How well learning methods generalize beyond the test sets of benchmark datasets has been getting attention in the fields that are pushing the supremacy of machine learning in pattern recognition. In computer vision research, object detection models trained on CIFAR-10 and ImageNet were tasked with evaluating images from reproduced CIFAR and ImageNet data generation experiments. The results showed a significant loss of generalized performance (SoTA ImageNet models lost 3–15% accuracy, SoTA CIFAR-10 models lost 11–14% points in accuracy) [16]. The fragility of natural language models (NLP) has been demonstrated several times, mostly to temper the claims that NLP models are outperforming humans in tasks such as question answering and translation [17,18,19].

#### 1.3.2. ML-NIDS Ignores Generalization

ML-NIDS eagerly adopts new techniques and model architectures from CV and NLP research but without validating general performance [20,21,22].

This can be explained, in part, by the sparse dataset landscape. The lack of ample, qualitative data in network intrusion detection research has been criticized frequently in the literature (2015: [23], 2016: [24], 2019: [25]). Central issues include the frequency of publication, the closed-off nature of the experiment and the dataset preparation, the variety and recency of included attacks and the realism of the user simulation. Two additional critiques that stand out to us include the relevance of the included features and the lack of consistency between datasets in terms of feature set. This restriction has kept everyone from investigating the halfway solution of model generalization between academic datasets.

Mentions of generalization in intrusion detection are either limited to theoretical considerations during algorithm choice [26] or mentioned as an abstract benefit that should follow from using classifier ensembles [27] or preprocessing. Furthermore, generalization in those articles is often synonymous with test set error (i.e., standard intradataset generalization). Testing generalization in tougher circumstances is only ever implied.

#### 1.3.3. Noteworthy Theoretical Objections to ML-NIDS

The potential for machine learning IDSs to overpromise and underdeliver in terms of general ability has been pointed out by proponents of rule-based systems. Perhaps foremost in a paper co-authored by one of the original inventors of Bro, currently Zeek IDS, which uses deep packet inspection to detect specific threats. Sommer et al. [28] wrote in 2010: “It is crucial to acknowledge that the nature of the domain is such that one can *always* find schemes that yield marginally better ROC curves than anything else has for a specific given setting. Such results however do not contribute to the progress of the field without any semantic understanding of the gain.” The authors argue in favor of an evaluation methodology that takes more into account than metric gains on benchmark datasets. At various points in the text, they remind the reader of the contrast between academic intrusion detection research which works toward a silver bullet solution and the collection of highly specialized tools that protect current networks.

Gates et al. [29] challenged anomaly-based intrusion detection at a more fundamental level. When network intrusion detection started, modeled on the requirements set out in 1985 for host-based intrusion detection systems, some issues were not considered carefully enough. The authors criticize nine assumptions, grouped into three categories: problem domain, training data and operational usability. Central issues of the problem domain are the assumptions that attacks will differ substantially from normal traffic, attacks are rare enough to be a small, but discernible cluster when compared to normal traffic and that anomalous traffic is much more likely to be malicious. The three prerequisites for training data are that there is attack-free data available, which is representative and static. Exceedingly few publications address concept drift in intrusion detection directly [30]. If the term is included, it is mostly lip service. A final category that is nearly absent in academic articles on intrusion detection is operational usability. Gates and coauthors point to realistic settings for parameters such as the false alarm rate. Even at 1% with potentially thousands of flows per second, this quickly becomes unmanageable. Other operational problems include the company policy towards what is malicious and the interpretability of anomalies by human reviewers. Ultimately, the authors made four recommendations which could be moved straight to the contemporary discussion. The authors recommend hunting for specific types of threats by a combination of classification and anomaly detection with testing methodologies that are much more rigorous than just using benchmark datasets and periodically re-examining what constitutes malicious behavior.

## 2. Materials and Methods

The methodology section is split in two parts. Section 2.1 and its divisions supply the reader with more information about the datasets. Section 2.2 elaborates on the intra- and interdataset evaluation frameworks, the included algorithms and the data reduction measures that had been applied in the previous work [15] to yield equally discriminative models with less (qualitative) data.

### 2.1. Included Data Sets

The sparsity of the dataset landscape in intrusion detection has been a continuous issue exemplified by the positions in the literature of KDD99, published in 1999, and NSL-KDD, a KDD99 refresh from 2009. These datasets have become fixtures with novel publications still using them as the central dataset(s), despite damning but valid criticism [31,32].

The one-off nature of these dataset creation experiments as well as their proprietary methods of data collection and preprocessing have barred the addition of new samples that reflect more modern threats. The tendency of singular efforts that culminate in one published dataset progressed throughout the past decade with datasets such as UTwente2009 [33], CTU-13 [34] and UNSW-NB-15 [35].

The Canadian Institute for Cybersecurity, a collaboration between academia and public/private sector partners, headed by the university of New Brunswick, advocates for a more dynamic and open approach to dataset creation. Three years after refreshing KDD99 [36], they published an initial dataset (ISCX-IDS2012), with inclusion of the ISCXFlowmeter feature extractor [37]. Although this dataset did not gain much traction with other researchers, it laid the groundwork for their current data publication schedule which has produced four IDS datasets in the last three years. Each entry includes the raw traffic and the labeled flows in CSV format. The latest version of their flow reconstruction and feature extraction tool is publicly available on https://github.com/ahlashkari/CICFlowMeter (accessed on 28 December 2022) Github. Sharing the feature extraction tooling has at least opened the door to other researchers to evaluate novel samples from new dataset generation experiments in proposed models, validated on the CIC’s datasets. A strong case can be made for open sourcing other parts of dataset creation methodology. Because this level of access is presently not available, this work focuses on working with the four recent CIC datasets (CIC-IDS2017 [38,39], CIC-DoS2017 [40,41], CSE-CIC-IDS2018 [42,43] and CIC-DDoS2019 [44,45]).

#### 2.1.1. CIC-IDS2017

CIC-IDS2017 (Table 1) includes a large variety of attack categories, with flows from multiple specific attacks per category. The malicious traffic is offset by a larger corpus of benign traffic from a multitude of protocols (HTTP(S), IMAP, SSH, FTP,...). The experiment was executed in an on-premise network environment over a period of five days. A split was made into seven distinct attack categories. There is an eighth split, which only contains benign traffic. Representationwise, most modern attack types are accounted for. Network-centric attack types such as (D)DoS, port scanning and brute force attempts are included, as well as more host/application-centric attack types such as web and infiltration attacks and botnet infections. CICFlowmeter extracts more than 80 features, of which some have to be removed due to them being meta-information to assist in bookkeeping and labeling which would contaminate models if included. The remaining 76 features typically characterize the network flows by way of aggregate statistics. Some examples include fl_iat_avg (average time between flows), pkt_len_avg (mean flow length) or fw_win_byt (number of bytes sent in the initial window in the forward direction). For a full feature list with short descriptions, consult the authors’ documentation [43]. The imbalance of this dataset for some attack types should not be ignored. For CIC-IDS2017 the ratio of benign to malicious ranges from 1.25:1 to 8000:1. Unsurprisingly, the network-centric classes have better class balance. These attacks are easier to execute and often naturally generate more traffic because of the mechanisms they exploit. The classification metrics in this text incorporate this imbalance, by making use of balanced accuracy to represent the whole and measures such as precision, recall and F1-score which give information about the classification of the positive (i.e., malicious) class.

#### 2.1.2. CIC-DoS-2017

CIC-DoS2017 (Table 2) is a one-off intermediate dataset that focuses solely on application layer denial of service attacks (DoS). The L7-DoS attacks in CIC-DoS2017 are a superset of the HTTP-DoS attacks in CIC-IDS2017. Application layer DoS attacks do much more damage with limited bandwidth. They typically abuse one of three mechanisms: slow send, maximally intermittent send or web server specific one-shot attacks. CIC-DoS2017 contains malicious traffic from eight different L7-DoS attacks, run over a 24 h period against 10 Web servers. CIC-DoS2017 has a class imbalance ratio of 5.8 (benign) to 1 (malicious).

#### 2.1.3. CSE-CIC-IDS2018

CSE-CIC-IDS2018 (Table 3) is a direct expansion of CIC-IDS2017. The on-premise network setup was scaled up on a cloud provider’s equipment (Amazon Web Services). More traffic was captured, with a focus to ameliorate the class imbalance of CIC-IDS2017. A total of 10 subsets were captured over a 10 day period. Some of the attack types have been split up into their own subsets while others have merged. The benign traffic has expanded in volume, but not in protocols whereas the malicious traffic has expanded in both directions. Some subsets (i.e., attack classes) now have an over-representation of malicious traffic. The only attack class which has regressed to lower representation compared to CIC-IDS2017 is web attack traffic.

#### 2.1.4. CIC-DDoS-2019

CIC-DDoS2019 (Table 4) focuses exclusively on reflection and exploitation-based distributed denial of service attacks. Akin to CIC-DoS2017, the end result is a dataset with a superset of DDoS attacks, compared to their representation in the full IDS datasets. Most specific types have representation in two designated subsets. Researchers who wish to improve DDoS modeling have an easy separation between training/validation samples and testing samples. Separate sets designated for training and testing, provided by the authors, have the added benefit of providing an even more stable reference point for adopters of the dataset. Because this work does not do training on CIC-DDoS2019, all subsets were used individually to assess the generalization potential of DDoS models trained on the CIC-IDS2018 dataset. The original class imbalance between benign and malicious samples of CIC-DDoS2019 was so egregious that all sets have been balanced by including all available malicious samples and a randomly sampled set of benign traffic from the same subset to match the malicious count.

### 2.2. Training & Evaluation Procedure

The intradataset framework is directly taken from the previous work that pushed the efficient use of training data and learning difficulty for 12 learning algorithms on four intrusion detection datasets (NSL-KDD, ISCX-IDS-2012, CIC-IDS-2017, CSE-CIC-IDS2018) [15]. The interdataset framework for pretrained models reuses the preprocessing and performance measuring from the intradataset framework. The maximal reuse of components is a conscious choice because changing design choices between the frameworks would introduce doubt about the influence of methodological changes as the source of the unexpectedly low generalized performance.

#### 2.2.1. Included Algorithms

The CIC-IDS2018 collection of pretrained models includes twelve supervised classifiers. Three classical algorithmic families are represented. The seven tree-based learners include multiple improvements to the base decision tree model. All but plain decision trees are ensemble methods that include bagging, some of which add boosting mechanisms and/or rely on randomization for efficiency and robustness reasons. The other five methods fall into two simple neighbor-based algorithms, two support vector machines and logistic regression. The inner workings of these algorithms are described in detail in the following references: [46] (general) [47] (xgboost framework) [48] (randomized decision trees). The listing includes abbreviations which will be used throughout the remainder of the article and the graphs.

1. Tree-based methods
  - Decision tree (dtree)
  - Decision trees with bagging (bag)
  - Adaboost (ada)
  - Gradient-boosted trees (gradboost)
  - Regularized gradient boosting (xgboost)
  - Random forest (rforest)
  - Randomized decision trees (extratree)
2. Neighbor methods
  - K-nearest-neighbors (knn)
  - Nearest-centroid (ncentroid)
3. Other methods
  - Linear kernel SVM (linsvc)
  - RBF-kernel SVM (rbfsvc)
  - Logistic regression (binlr)

#### 2.2.2. Increasing the Learning Difficulty

As alluded to earlier, when classifying within the included datasets, all models showed great resistance against the two major forms of data reduction. Vertical data reduction, i.e., setting increasingly stringent constraints on the amount of samples made available to train/validate with the complement of samples for testing, could be pushed to extreme splits such as 0.66%/0.33%/99% without incurring significant losses. There was some variation between models and attack classes but stable convergence of performance on intradataset test sets always occurred before using 10% of the data for training/validation. Any sampling happened in a stratified (i.e., respecting class balance) manner. To increase the learning difficulty even further an opposite approach to the standard feature selection procedure was adopted. The top 20 features, derived from averaging the feature importances of the aggregate of random forests for CSE-CIC-2018, is listed in Table 5. Instead of removing the less important features, the 20 most important features were removed in blocks of five prior to training. This had a greater effect than vertical data reduction, but the easy (network-centric) attack classes were still barely affected. Along with an invariance to feature scaling and clear stability with small metric deviations from the mean after aggregation in a fivefold cross validation procedure, the experiment concluded positively about using classical supervised ML methods in intrusion detection.

## 3. Results

The following subsections summarize and highlight the important results of this analysis. First, the base recognition of the models trained on the attack classes in CSE-CIC-IDS2018 is established Section 3.2. The first information on generalization consists of the ability of models pretrained on classes for which two subsets of traffic were available, how well they generalize to each other’s data. Section 3.3 is an expansion of this analysis to all available attack classes and moves across the boundaries of a single dataset. The last two subsections (Section 3.4 and Section 3.5) delve deeper into the generalization capabilities on two of the best-recognized classes DoS and DDoS by exposure to two specific datasets that contain a larger variety of attacks from these classes. Discussion and linking is interwoven in the content of the subsections (and their further subdivisions), but to keep an overview per dataset, every subsection ends with a minor discussion/conclusion. Some specific issues are only visible when examining recurring patterns in the dense result collection. To maintain an overview of the potential of the included algorithms in IDS systems, Section 4 gives a much less verbose view of the best results.

**Figure 1:**
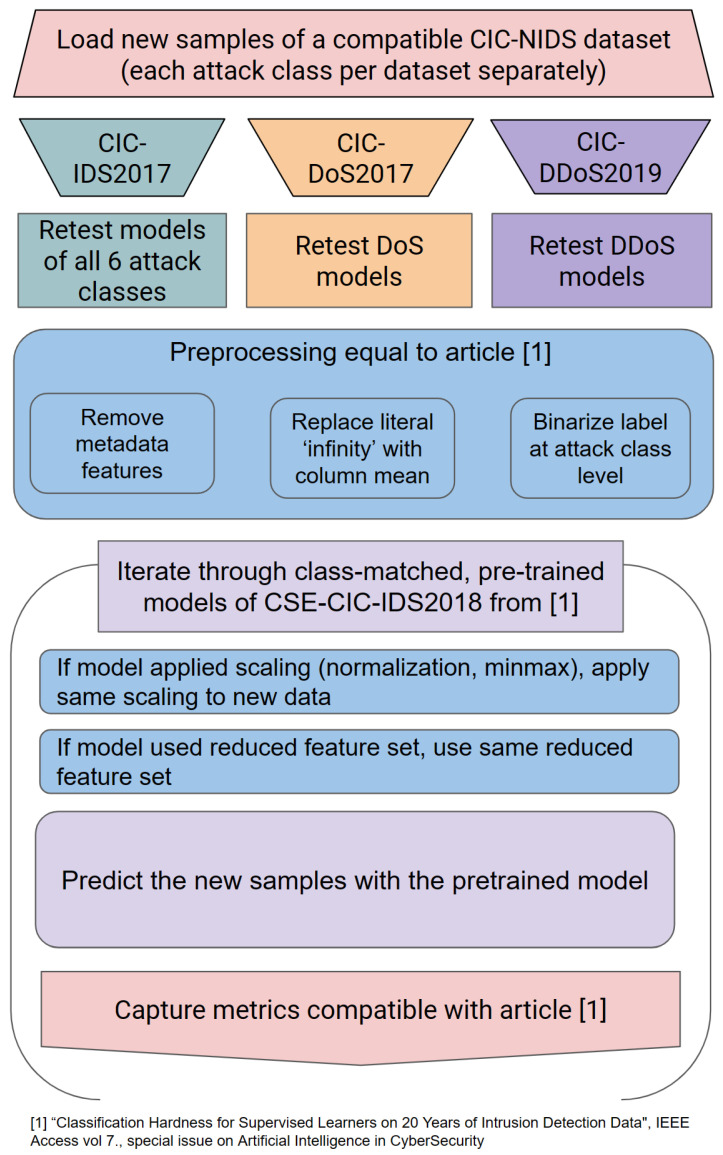
Visual overview of the experimental methodology.

Figure 1 provides a visual overview of the methodology to be able to reproduce this experiment more easily.

### 3.1. Note on Obtained Results and Graphics

The visualizations in this article are in support of the most salient results. The total amount of available, interactive graphs however is much larger (covering more than half a million result metrics). All raw result files (grouped per algorithm in folders D2018-M2018, D2017-M2018, D2017DoS-M2018 and D2019DDoS-M2018) and plotting code is publicly available at https://gitlab.ilabt.imec.be/lpdhooge/reduced-unseen-testing (accessed on 1 February 2023). With transparency and reproducibility in mind, the same repository also contains the experiment code.

### 3.2. Internal Retest on CSE-CIC-IDS2018

To establish a baseline, the newly trained models were re-exposed to all data of the subset on which they were trained. For models trained and validated on larger portions of the data, this means that more samples that had been seen during training will be re-evaluated. This might introduce some skew in the results. For models trained and validated on little data, i.e., <5% of the samples, this is less of an issue.

This subsection will contain new pieces of information as well because CSE-CIC-IDS has multiple instances where two subsets of traffic from the same class have been collected. Therefore, it is possible to test generalization within CSE-CIC-IDS2018 between models trained to classify the same attack class. The DoS (subset 1 and 2), DDoS (3 and 4), Web attack (5 and 6) and infiltration (7 and 8) attack classes each have two subsets. For the Web attack and infiltration classes the documentation remains vague on what the differences between the two subsets are, so a substantial amount of overlap should be assumed. This overlap is more clearly stated for the DoS and DDoS classes, where some tools to generate attacks are used in both subsets and some are different.

#### 3.2.1. FTP/SSH Brute Force

Tree-based models as well as knn, linsvc, binlr and rbfsvc reach perfect classification scores. Both relationships training volume—classification performance and top-feature removal classification performance behave as expected with an upward trend when increasing training volume and a flat reduction in classification scores when disallowing the models to use the most discriminative features.

#### 3.2.2. OSI Layer 7 Denial of Service

**Figure 2:**
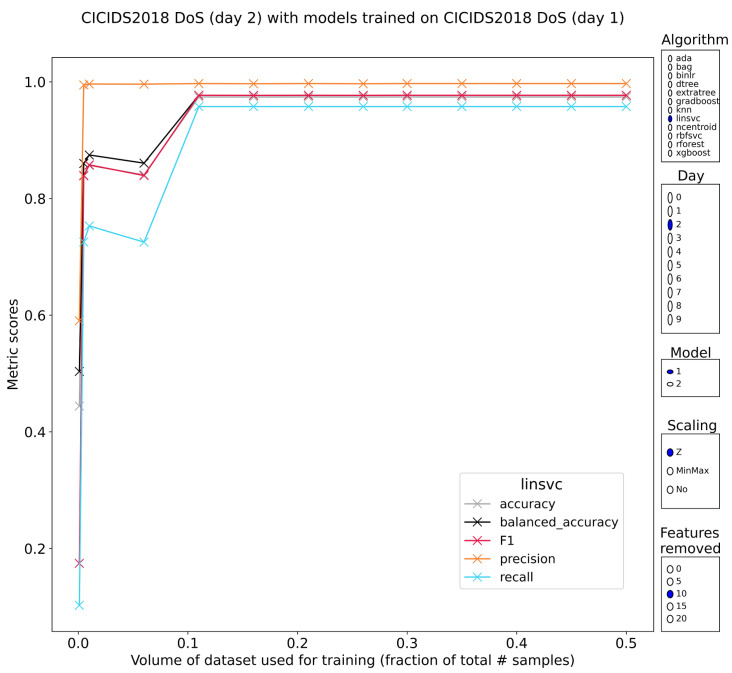
Performance profile of the set of linear SVMs, trained on the first subset of DoS traffic in CSE-CIC-IDS2018, when evaluating the second DoS subset from the same dataset.

Layer-7 denial of service (DoS) attacks are extremely well-classified by all models except nearest-centroids. F1-scores above 99.5% even for models trained and validated on just 0.1% (associated 99.9% test) of the data are the norm even when removing the 25% most discriminative features prior to training. Unfortunately, when feeding the benign and malicious samples of the second subset of DoS traffic to models trained on the first subset and vice versa, these perfect scores do not remain. By and large the models revert to balanced accuracies of 50%, which indicates models that are not better than chance at recognizing the attacks. The new F1-scores mostly stay below 10%, showing that the classification of attacks is lackluster. The confusion matrices for the individual models confirm this, showing that most of the errors are type II (false negatives). Learning with regard to training volume is nonexistent. There is one glaring exception to this crash in classification performance. The linear SVMs trained on the normalized features in subset 2 of DoS traffic generalize as expected to the samples in DoS subset 1 (Figure 2). F1-scores above 97.5% are observed for all models that used more than 5% of the data to train. The results are stable and top-feature reduction has an impact, but it is minor.

#### 3.2.3. Distributed Denial of Service

**Figure 3:**
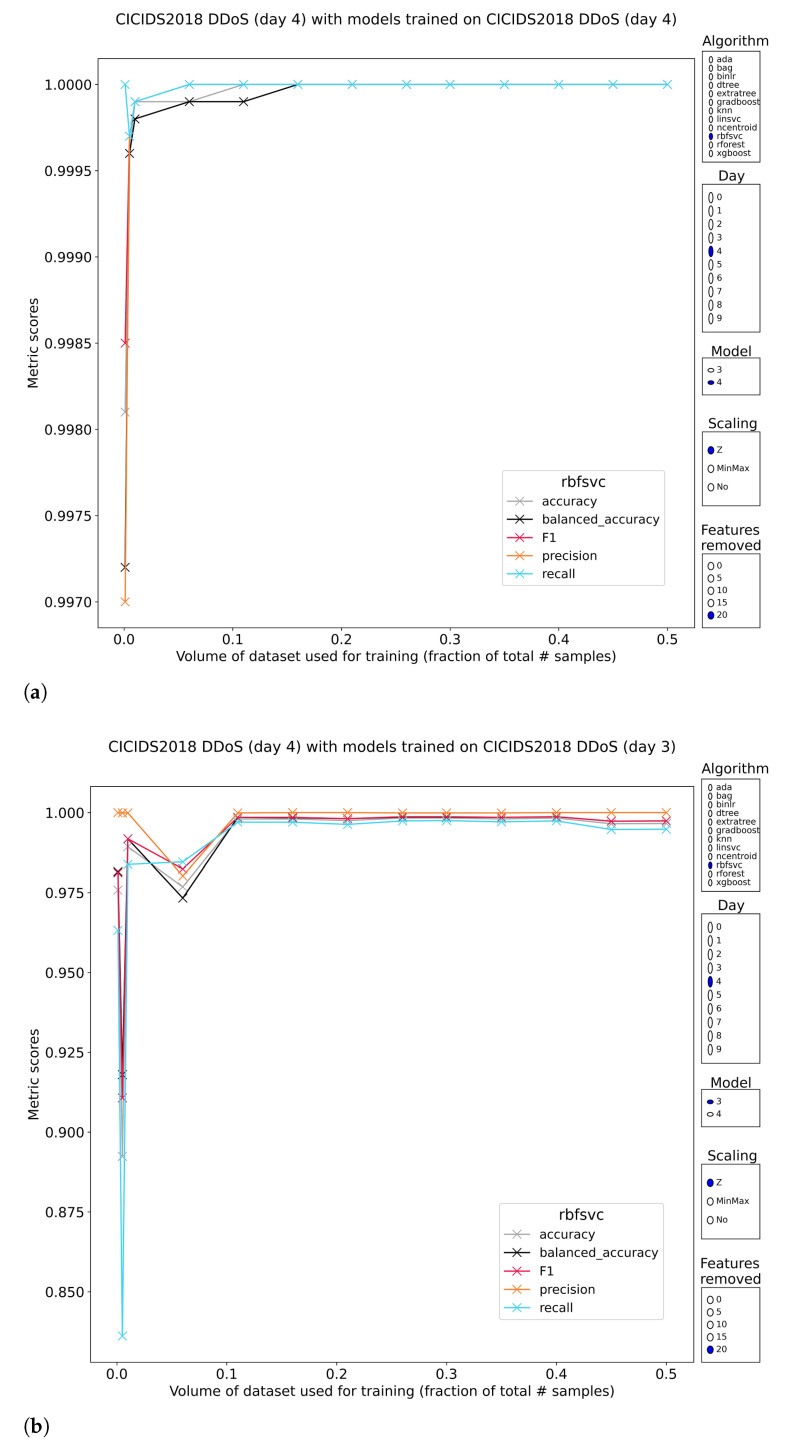
Performance comparison of the set of RBF-kernel SVMs, trained on the second subset of DDoS traffic in CSE-CIC-IDS2018, when evaluating its own test set (**a**) and the same set’s performance when evaluating the samples of the first DDoS subset of the same dataset (**b**).

Distributed denial of service (DDoS) attack samples (subsets 3 and 4) are as well classified as Layer-7 DoS samples (99.9%+ on all metrics, with fast conversion to an upper bound with <5% training volume and resistant to the removal of the most discriminative features). The results of exposing the pretrained models to the other subset containing DDoS samples paints a different picture of the classification performance. While it is possible to achieve models that have generalized perfectly to the class, this happens rarely (Figure 3). The strongest performers are the SVMs and logistic regression models. Tree-based models struggle by overfitting almost immediately. This is clearly visible in the result graphs because invariably, the best tree-based models have only been trained on less than 1% of the data. These models can reach generalized performance up to 80% on the F1 score. In cases where precision is high, it is most often 100%. The associated recall keeps these methods down.

#### 3.2.4. Web Attacks

Web attack traffic is harder to classify, as evidenced by the lower overall classification scores for all algorithms. The tree-based methods converge to 95% classification metrics but take more training data than they did to accurately classify the previous attack classes. They are now also significantly weakened when removing the most discriminative features. Methods such as nearest centroids, binlr, linsvc and rbfsvc have such poor precision scores that they are not even usable. Subsequently, pretrained models based on these methods are not expected to do well when evaluating the samples of the other subset. The experimental results confirm this with similar classification profiles (~0% precision regardless of training volume or feature scaling). The tree-based methods fare much better, but for this behavior to emerge the models had to be trained on nonscaled features and none of the most discriminative features should have been removed from the data prior to training. Those parameters lead to F1-scores that are consistently in the 80–85% range (class balanced accuracy 95+%). The behavior of these models when removing top features is as expected, showing a flat drop in classification performance (up to −35%), but stability is retained. For tree-based methods this stability is peculiar, but it is an essential property if they are to be included in real IDSs. This result does show that it is possible to train stable tree-based methods, however further inquiry is needed to specify what the training conditions should be. As stated in the introduction to this section, the Web attack class has two separate subsets of traffic, but a substantial overlap in the attack methods should be assumed as per the documentation of CSE-CIC-IDS2018.

#### 3.2.5. Infiltration Attacks

**Figure 4:**
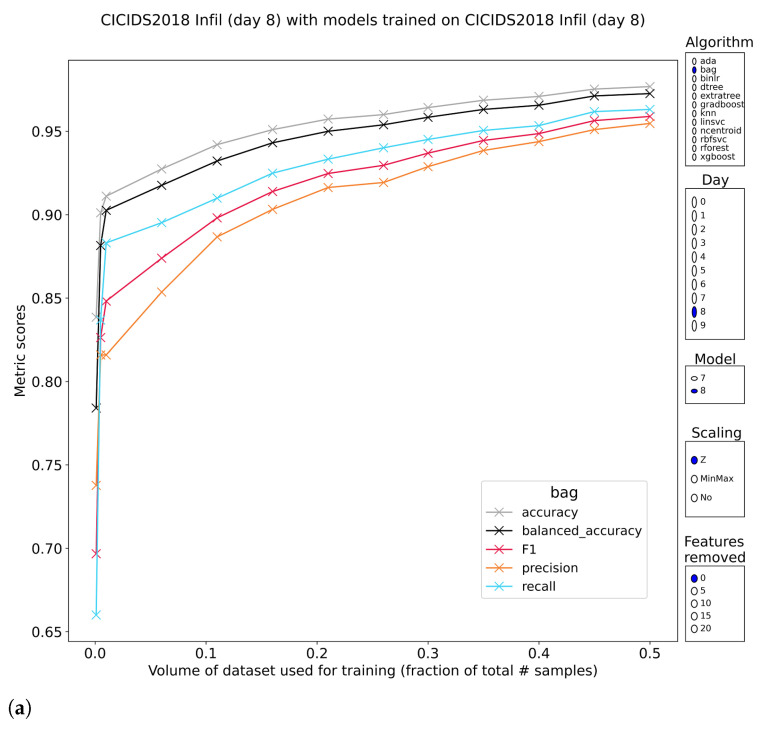

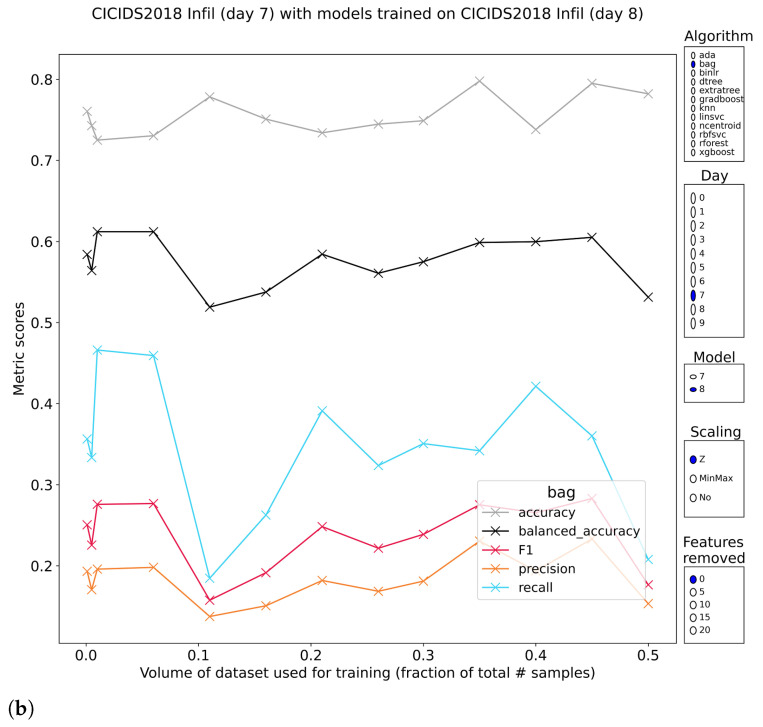
A visual representation of the broken link between training volume and model performance, observed in the breakdown of models trained on subset 2 of infiltration traffic (**a**) when classifying their subset’s test set and the same set of models’ performance on subset 1 of infiltration traffic (**b**).

Recognition of infiltration attacks is poor for all methods on the first subset (7) but substantially better for subset two (8). The documentation of CSE-CIC-IDS2018 does not state how the infiltration attacks were executed in both subsets, but based on the classification scores, it is safe to assume that the methodology differs. In concrete numbers, classification metrics on subset 7 typically only have F1-scores below 50%. F1-scores on subset 8 reach between 85–95% for the tree-based methods, with the other methods trailing significantly behind at around 50% (F1). The models with decent performance are also stable with regard to training volume, showing an upwards trend when allowed more data to train on. Furthermore, this stability extends to models that were trained without access to the most discriminative features. As expected, the models pretrained on subset 7 that all performed poorly overall have poor performance when classifying the samples of subset 8. The other half with models pretrained on subset 8, evaluating the samples from subset 7, reach a conclusion that is much more interesting. That conclusion is a performance regression to low levels, indicating that the results obtained on the subset itself are misleading. A learnable pattern does exist in the data, but it is not descriptive of the difference between regular traffic and infiltration attacks (Figure 4). F1-scores most often drop to 15–30%, rendering the models useless for practical purposes. Results like this show why it is needed to validate existing models on novel but related samples because omitting that part of the analysis would lead to the conclusion that the methods are suitable. This conclusion would have been reinforced by the stability of the outcomes with regard to training volume and feature reduction.

#### 3.2.6. Botnet Attacks

The final class in CSE-CIC-IDS2018 contains botnet traffic from two different botnets (Zeus [49] and Ares [50]). The dataset contains all samples grouped in the same subset (9). The labeling in the data prohibits manual splitting to execute analysis akin to that of the previous subsections, using only botnet as label as opposed to keeping the distinction between Zeus and Ares. Therefore, this subsection can only establish the baseline that should be considered as the starting point for comparisons when exposing the pretrained models to the botnet data of CIC-IDS2017 (Section 3.3.5). The baselines are that the tree-based methods reach perfect classification with little data to train (~1%), losing at most 0.5% when reducing the 20 most discriminative features before training. The binlr, linsvc and rbfsvc models do not quite reach perfect classification with F1-scores consistently above 90% even when allowing little of the data to be used as training samples.

### 3.3. CIC-IDS2017

This section details the important experimental results of tasking the class-specific models trained on CSE-CIC-IDS2018 with classification of the corresponding attack class of CIC-IDS2017. To maintain oversight, Table 6 lists the mapping between the pretrained models and the new data which they will have to classify. There are lots of similarities in the methodology by which both datasets have been created. The same attack classes are represented in both datasets, often generated with the same tooling. This should be advantageous for the pretrained models. One important point of difference however is the move from on-premise equipment to running the experiment on Amazon Web Services, which has introduced changes in the network architecture and thus potentially in the base network patterns. Whether this factor has significant implications and, if so, how they should be quantified remains an open question.

#### 3.3.1. FTP/SSH Brute Force

Subset 0 of CIC-IDS2017 contains attacks generated by the Patator tool against an FTP and an SSH endpoint. Patator is designed as a multipurpose brute-forcer, a general framework to execute brute force attacks against an array of services (https://github.com/lanjelot/patator accessed on 28 December 2022).

**Figure 5:**
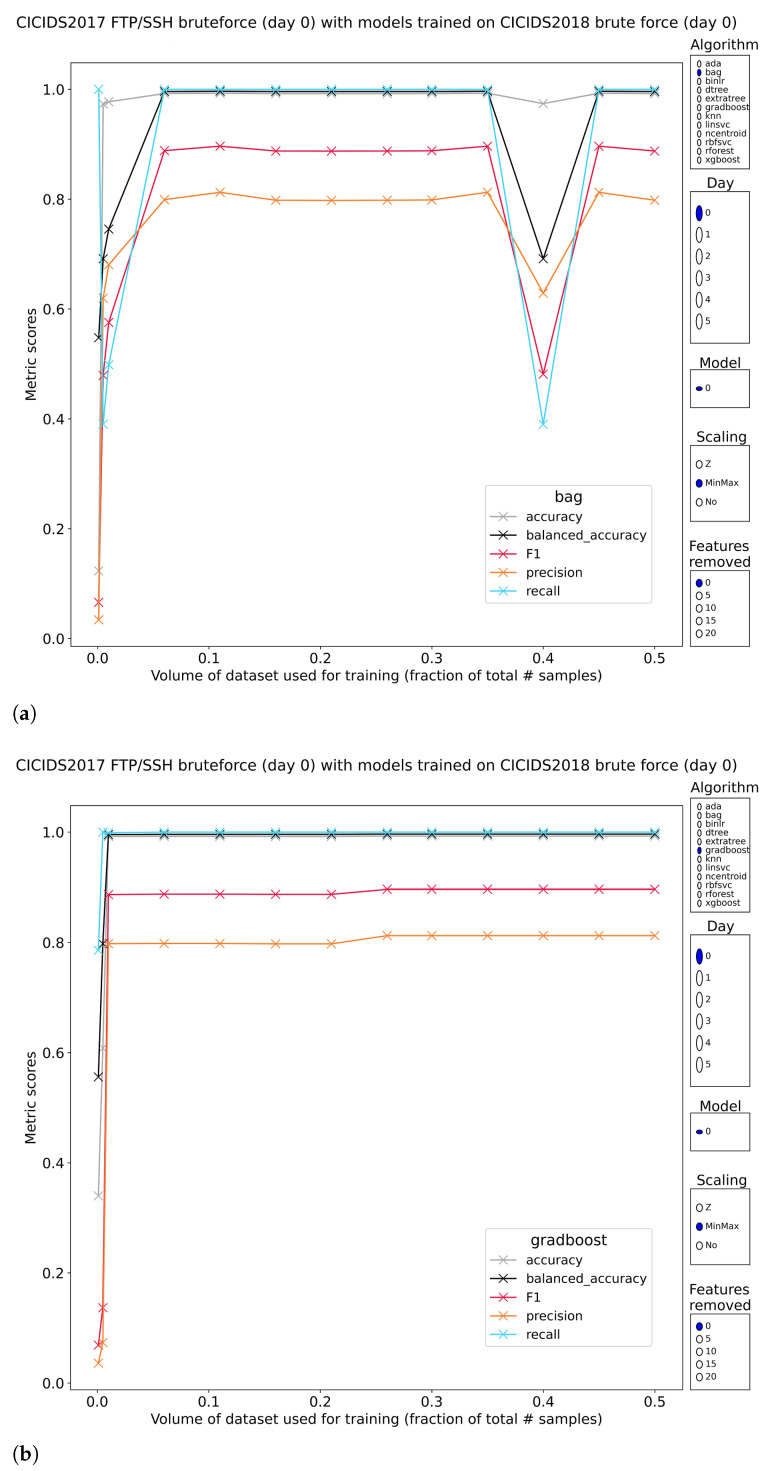
Best-case generalized performance of tree-based ensemble classifiers trained to recognize bruteforce attacks from CIC-IDS2017, when evaluating the bruteforce samples of CSE-CIC-IDS2018.

Results of the tree-based classifiers reveal that generalization was possible under the right circumstances (Figure 5). While no longer perfect at classification, the methods preserve up to 89% F1 score (recall 99.9+% with 81.5% precision). The mistakes made by the models are almost exclusively type II errors. The single combination of pretraining parameters that yielded models with good results is that they needed access to all features and were minmax-scaled before training. Any deviations from this result in models that have F1-scores in a range as low as 0–10%. The models with good performance did not have perfect stability with regard to training volume. All methods contain at least one sharp downturn in performance, happening at an unpredictable training volume.

The neighbor-based methods are too simplistic with F1-scores in the 10–20% range, invariant to training volume or feature removal. The other methods are not usable either with similar classification scores.

#### 3.3.2. OSI Layer 7 Denial of Service

CIC-IDS2017 has bundled all (application layer) DoS attacks in a single subset, whereas CSE-CIC-IDS2018 split this into two hence two pretrained models have been tested. The obvious result is the loss of perfect classification and the introduction of dramatic performance swings. The fork in which metrics such as the F1-score lie has a top end above 90% and a bottom as low as 0%. The relation between training volume and performance is not predictable, further complicating the matter. The models pretrained on DoS subset 2 of CSE-CIC-IDS2018 are consistently worse than those trained on subset 1, which is surprising given that CIC-IDS2017 is actually biased toward the attacks contained in DoS subset 2 of CSE-CIC-IDS2018. The models that exhibited decent performance required either minmax- or no feature scaling prior to training. Removal of top features cripples these methods further to the point of irrelevancy.

**Figure 6:**
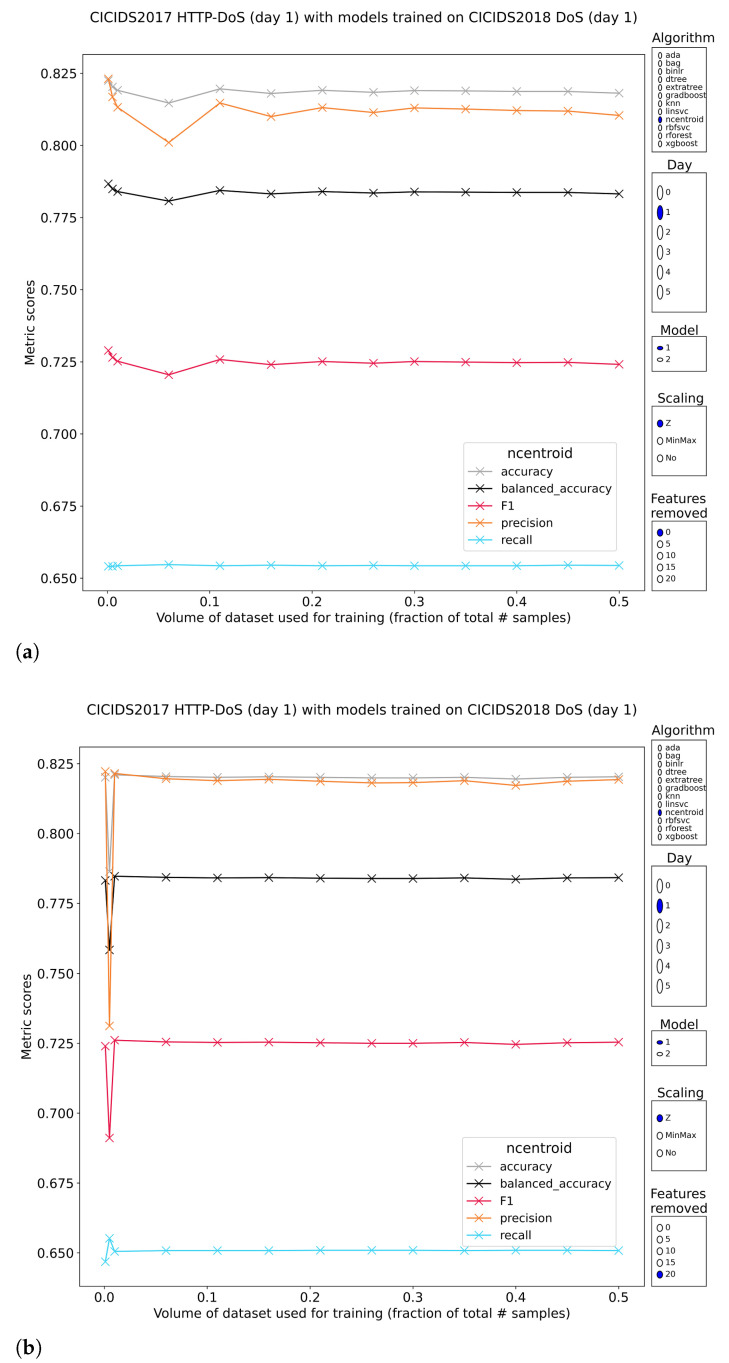
Generalized performance of the nearest centroids models when evaluating L7-DoS attacks between datasets and their resistance to the removal of highly discriminative features prior to training.

Curiously, the simplest method, nearest centroids, is quite capable of distinguishing between DoS and normal traffic (Figure 6. In absolute figures, it reaches F1-scores around 72.5% (models pretrained on CSE-CIC-2018 DoS subset 1). Extra desirable properties include the invariance of these scores to training volume and/or feature reduction. A stratified sampling of just 1% in volume to train and validate is enough to produce the outcomes. Resistance to removing some of the most discriminative features worked best if the features were normalized before training, showing total disregard for the attempt at reducing the model’s effectiveness.

**Figure 7:**
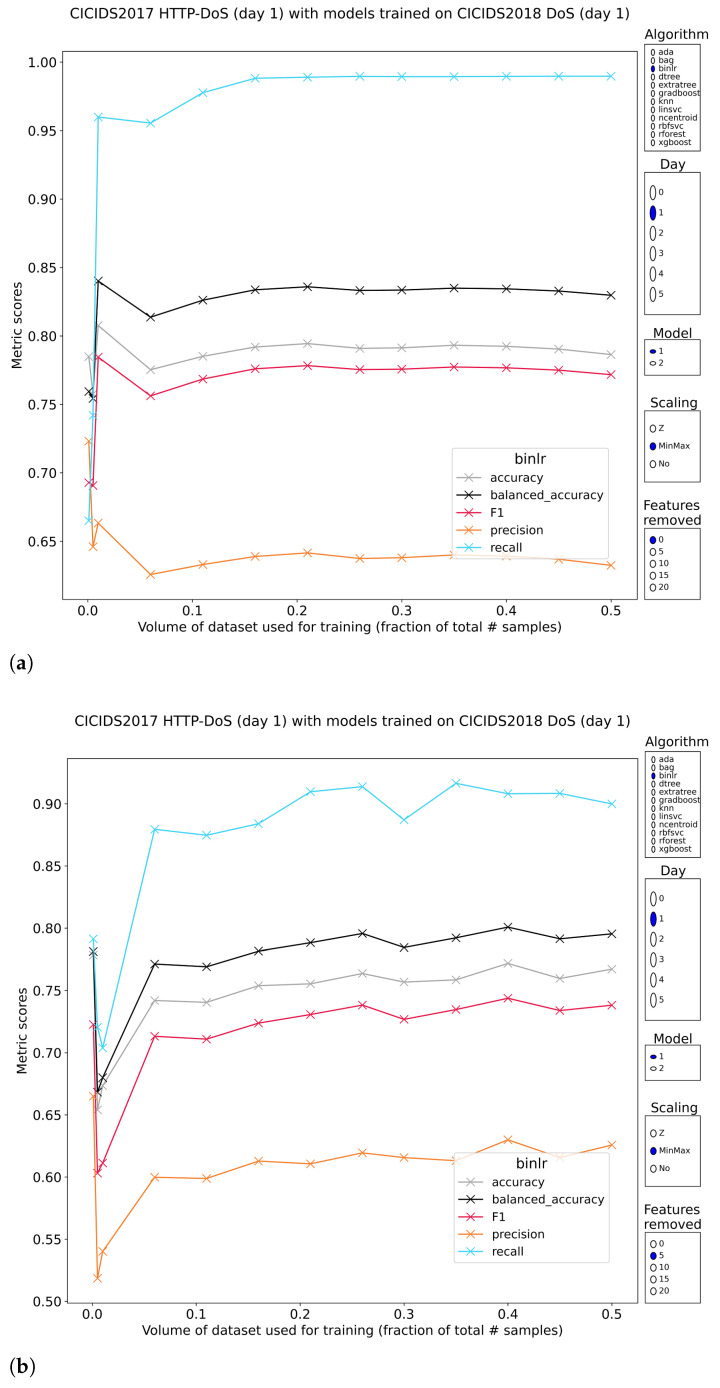
Generalized performance of logistic regression models on the L7-DoS attack class, which show reasonable consistency.

The other methods provide the first set of consistent models with somewhat acceptable performance. The logistic regression model trained on minmax-scaled features, is stable in relation to training volume and has F1-scores up to 78% (Figure 7). It would be a stretch to label this performance as good, but the stability is an interesting property. Similar conclusions can be drawn from the results for the linear SVM and its rbf-kernel counterpart. These methods have the additional benefit of withstanding reduction of top features up to a common breaking point. These properties are essential to dependable training of real intrusion detection systems. The lackluster precision (~65%) that keeps these methods down, might be elevated to a higher level by applying the feature reduction/selection techniques in their standard direction, cutting out the irrelevancies and the noise.

#### 3.3.3. Web Attacks

The crossover testing with pretrained models within CSE-CIC-IDS2018 revealed that generalization did happen for tree-based models if training was done on nonscaled features and without the removal of the most discriminating features (Section 3.2.4). Results with other parameters were not worthy of further consideration. Looking at the results for generalized performance on the Web attack traffic of CIC-IDS2017, a different picture emerges. There is not a single pretrained model that performs well on these samples. Even the distance-based methods now generate exclusively false negatives, indicated by a balanced accuracy of 50%. The models are not better than chance at classifying any of the Web attack traffic. This is especially strange because the documentation of both datasets mentions very similar attacks. It calls into question if the stable generalized results within CSE-CIC-IDS2018 Web traffic are actually the models learning a stable pattern that is unrelated to Web attack traffic.

#### 3.3.4. Infiltration Attacks

Infiltration attacks are underrepresented in CIC-IDS2017 with only 36 infiltration samples out of a total of 288,602. This massive imbalance should be kept in mind when interpreting the generalization results. The intradataset generalization testing for infiltration attacks (Section 3.2.5) has shown that it was not possible to learn a good representation of infiltration traffic, with F1-scores dropping to 15–30%. The results on the infiltration traffic of CIC-IDS2017 are even worse, because none of the methods reach F1-scores above 0%. The extremely poor performance is caused by the complete inability of all methods to be precise when classifying the samples. Moderately high recall can be observed (~80%), but coupled with 0% precision, this is not useful.

#### 3.3.5. Botnet Attacks

**Figure 8:**
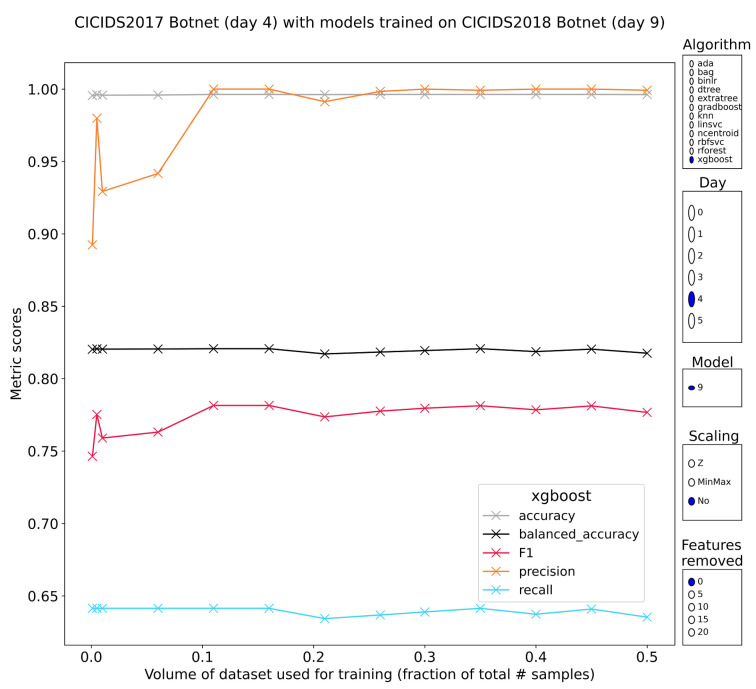
Best-case generalized performance by gradient-boosted, regularized decision tree ensembles on the botnet class.

CIC-IDS2017 uses the ARES botnet to generate samples for the botnet class. CSE-CIC-IDS2018 adds another botnet, namely ZEUS. Since the models are thus pretrained on a larger variety of botnet traffic, the expectation is that the performance will be good. Upon inspection of the results, it can be shown that this claim is partly true. Once again, tree-based models trained without feature scaling and without feature reduction, perform rather well. F1-scores sit consistently in the 75–80% range (65% recall and 100% precision) with class separability at 82%. Methods such as adaboost, gradient-boosted trees and XGBoost (Figure 8), hold this performance stably, while the other methods have some sharp drops at unpredictable training volumes. Another weakness of these methods is their dramatic loss of classification performance when removing the most discriminative features. Performance immediately degrades to unusable levels with F1-scores around 0%. Strong resistance to feature removal was a characteristic of the same models when training and evaluating only within CSE-CIC-IDS2018. Once more, the desirable properties disappear rapidly, calling into question the overall usefulness of these methods.

#### 3.3.6. Distributed Denial of Service

The final class that is represented in both CIC-IDS2017 and CSE-CIC-IDS2018 is distributed denial of service. Both datasets rely largely on the same tooling to generate samples of this attack class. Section 3.2.3 details how the DDoS samples are recognized perfectly even with low training volumes and aggressive feature reduction. That strong baseline is immediately diluted by showing that the tasking classification of the 2018 pretrained models on each other’s data leads to performance that is significantly worse. In the end, generalization worked best for the SVMs and logistic regression models, while tree-based models could hold their own when trained on very low volumes to avoid overfitting.

**Figure 9:**
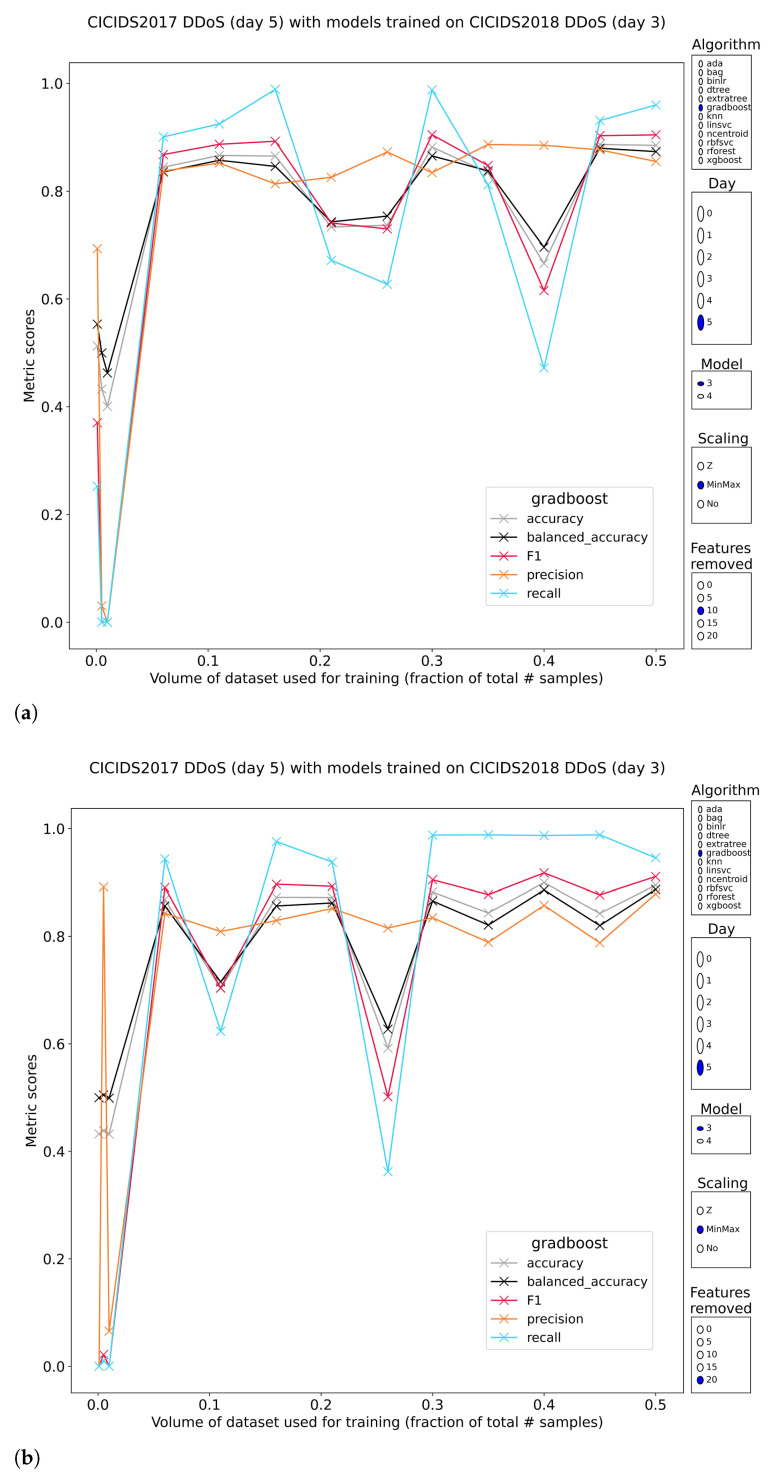
Generalized performance on the DDoS attack class by tree-based models can happen with modest losses in performance, but the unpredictability with regard to stability is a major downside (**a**,**b**).

When exposing the same pretrained models to this DDoS data, a similar conclusion can be drawn. Good to great generalization is possible, with tree-based methods as well as the logistic regression and SVMs. Even the nearest-centroids classifier performs reasonably (97.7% recall, 61% precision), despite summarizing each of the two classes into one label each. This shows that the feature values that are computed for the flows do differ significantly for DDoS versus benign traffic. Models with more complex means of separation classify the DDoS samples better. Here, the tree-based methods manage peak F1-scores of 90% (99% recall & 81% precision) and maintain these when removing the most discriminative features. Only two downsides remain: the occasional downward performance spikes and the lack of lockstep performance metrics between the models pretrained on subset 1 and subset 2 of 2018 DDoS traffic (Figure 9). These shortcomings make overall recommendations difficult. The tree-based methods that were trained on minmax-scaled features performed best overall while SVMs and consorts preferred normalized features. This is a rare instance where tree-based methods are robust to feature reduction and although stability is not yet at a level where these methods can be easily included in real IDS, the results are promising. Further investigation into how these tree-based models could be made more robust, will be paramount to advance.

#### 3.3.7. Intermediate Conclusion

Summarizing interdataset general performance from models pretrained on the attack classes of CSE-CIC-IDS2018 to samples from the same attack classes in CIC-IDS2017 is straightforward. True network attacks (brute force, DoS and DDoS) can be recognized with minor regressions in absolute performance. Botnet traffic sits at an intermediate level, and models for Web and infiltration attacks that just use the network for transport have really poor performance. Tree-based learners typically outperform versus the other learners on the classification metrics.

Absolute performance, however, is not the entire picture. Reliability is much weaker in the interdataset evaluation than it was for the exact same models during intradataset evaluation. As expected, the fragility is more pronounced for tree-based learners than it is for distance-based learners or regression models. Other properties that were lost in the interdataset results versus the intradataset results are the clear relation between training volume and model performance, the resistance against deliberate data quality reduction and the indifference to feature scaling.

In short, even with tight overfitting control during training and hyperparameter search, the excellent classification scores on the extensive test sets of CSE-CIC-IDS2018 cannot be trusted as one-to-one proxies for general model performance. Advances in model training and preprocessing may solve the reliability problem for network-centric attack classes, but the other attack classes will require other solutions first.

### 3.4. CIC-DoS2017

Letting the models pretrained on the DoS subset of CSE-CIC-IDS2018 classify the samples from the specific CIC-DoS2017 data set yields no satisfactory results. The overwhelming majority of pretrained models do not reach F1-scores above 40%. This is in spite of the fact that the malicious samples in the data set come exclusively from low-volume layer 7 DoS attacks. Combining the results described in Section 3.2.2 and Section 3.3.2 leads to an even more damning conclusion. Relatively speaking, layer-7 DoS is among the classes that the methods were able to best generalize to, but the results on this data set undermine the earlier results. The nearest centroids classifier has decent performance on some occasions (recall: 68%, precision: 52%). Tree-based models have recall–precision pairs that are most often separated by a large gap. Either precision is very high 80–100% with low to very low recall 0–30% or vice versa. The documentation that supports CIC-DoS2017, provides a clue about this poor performance. It states that the attack samples were intermixed with attack-free traces from the ISCXIDS2012 data set. ISCXIDS2012 was the first published iteration of a new intrusion detection dataset, after the CIC had published NSL-KDD. A potential explanation could be that the baseline traffic of ISCX-IDS2012 is more different from the newer baseline traffic that was generated for CIC-IDS2017 and CSE-CIC-IDS2018.

### 3.5. CIC-DDoS2019

The most recent dataset iteration published by the CIC focuses on DDoS attacks. It contains both reflection-based DDoS attacks as well as exploitation-based ones. As a rationale for the creation of this dataset around a specific attack type, the authors cite the small scope of attacks, covered under earlier taxonomies. In concrete terms, CIC-DDoS2019 contains 12 different DDoS attacks against a diverse collection of target machines (Windows Vista, 7, 8.1 and 10 as well as Ubuntu Linux 16.04). The raw packet captures as well as the labeled CSVs amount to a very large sample collection (more than 48 million of which only around 110,000 are malicious). In order to cut down on processing time, the files have been reduced to reach a balance between benign and malicious samples. Since the benign traffic far outstripped the attacks in volume, the reduction matches a sampling of normal traffic equal to the malicious count for every subtype of DDoS attack (Table 4). The discussion of the classification results will be divided along these subtypes. The models pretrained on the DDoS class of CSE-CIC-IDS2018 have only seen traffic generated by the Low-Orbit-Ion-Cannon tool (LOIC), which only generates UDP, TCP or HTTP floods. The attacks in this dataset span many more protocols, but if they are properly placed under the umbrella term DDoS, the models should be able to latch onto the general representation of DDoS traffic.

#### 3.5.1. Reflection-Based DDoS

The traffic in this category is generated by abusing genuine commands that exist in the implementations of several protocols. Attackers try to find a request that is small in size but has a large response, thereby achieving amplification. The secondary component to these attacks is executing them from a network that allows IP spoofing, redirecting the responses to an arbitrary IP. In lab settings, IP spoofing is not even required as ARP poisoning can redirect all traffic on a subnet to a specific host.

- DNS

**Figure 10:**
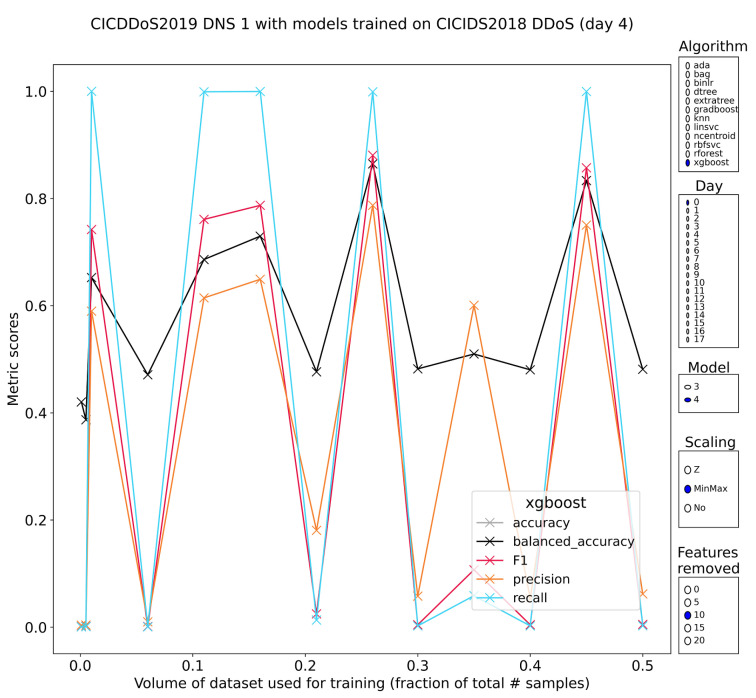
Generalized performance of DDoS models pretrained on CSE-CIC-IDS2018 DDoS has the potential to be high, but the volatility precludes reliability.

DNS amplification is a typical DDoS attack whereby spoofed clients send small requests such as ”dig ANY [domain] @[resolver] + edns = 0 + notcp + bufsize = 4096“ that lead to large responses (50×) sent over UDP. The requests are sent to open DNS-resolvers that respond to spurious requests. Some of the tree-based methods have very high performance with up to perfect recall, matched with 60–80% precision, but the volatility is staggering (Figure 10). Moreover, although there are two separate subsets of DDoS traffic in CSE-CIC-IDS2018, there is typically little agreement between the models trained on these separate subsets. Part of this might be due to the fact that the first subset has HTTP flooding attacks as well as UDP flooding attacks. It should also be noted that the tooling of CSE-CIC-IDS2018 for DDoS attacks is limited to pretty straightforward tools that do not use amplification.

- LDAP

**Figure 11:**
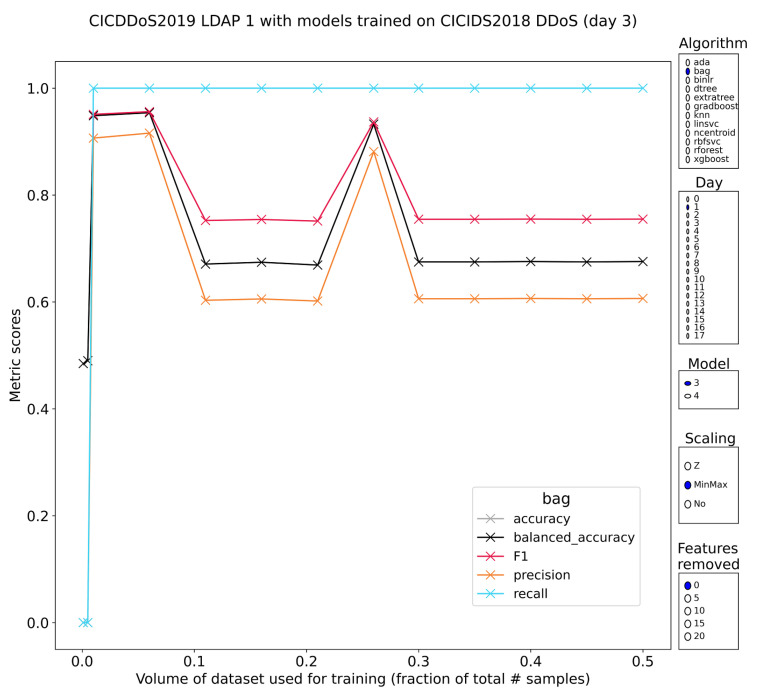
A rare occurrence of stable, generalized performance by a set of tree-based models. The best general performance exists for models that had little exposure to training data (<2.5% of sample count). This observation is very common, especially for the tree-based learners.

The subsets labeled 1 and 11 of CIC-DDoS2019 contain LDAP DDoS amplification attacks. The ingredients for this form of attack are similar. LDAP servers will typically communicate over TCP, but they offer a UDP endpoint as well. Well-configured LDAP servers should not respond to information requests by systems that have no authority to ask, but unfortunately, misconfigured LDAP servers exist that serve the open Internet. Services like Shodan scan the Internet looking for all kinds of vulnerable machines and subsequently expose this data through an open and a paid API. The tested algorithms show that they are capable of separating the novel malicious samples from the benign traffic with which they have been mixed and that separation can be done with a certain amount of stability (for example, Figure 11). Only the tree-based learners had acceptable performance and only if they had been trained on minmax-scaled features. The majority of models still did not have stable plateaus of classification performance but typically had an early spike (< 1% training volume) in performance (~85% F1), followed by a sharp decline. As with most of these models, the declines lead to very low levels of performance, mostly stably down, but unpredictable upshots do exist.

- MSSQL

**Figure 12:**
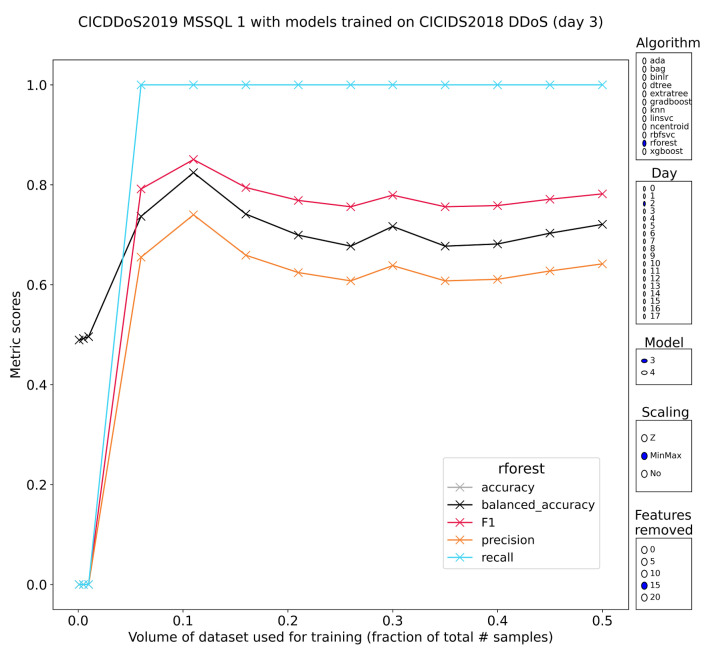
More observations of stable, general performance on the DDoS class by tree-based meta-estimators.

A discovery protocol for Microsoft SQL Server (MSSQL) that informs clients of the capabilities of an MSSQL cluster is vulnerable by design to be abused for DDoS purposes. It works on top of UDP and the functionality is to serve information to interested clients so attackers can blend in with legitimate users. Subsets labeled 2 and 12 from CIC-DDoS2019 contain samples of this attack class. Overall recognition here is once again done best by meta-estimators based on decision trees. A random forest trained on minmax-scaled features has the expected pattern with regard to training volume and maintains a stable profile even when removing the 15 most discriminative features from the data prior to training (Figure 12). These models performed equally well on subset 2 and subset 12. Such concordance between pretrained models is rare, but because the instances can be found, it must be possible to train the models to have a good general understanding of DDoS traffic. Simple methods such as knn or nearest centroids also performed quite well on the MSSQL traffic, reaching recall upwards of 90%, coupled with precision above 80% (knn) and 100% recall coupled with 50% precision (ncentroid). It should be noted that the reported classification scores only apply to models with minmax-scaled features.

- NetBIOS

The NetBIOS amplification attack abuses the NBSTAT query within the protocol that gives a status about an endpoint that responds to NetBIOS queries. Although the amplification factor is low at 3.8× [51], this attack has been observed in the wild. The results clearly indicate that the set of models that was dominant in previous subsections remains dominant on this form of amplified DDoS over UDP. The bagging classifier built on decision trees has stable performance with perfect recall and 61% precision, with the best model scoring 95.5% recall and 87% precision. Similar scores exist for the gradient-boosted trees (normal and regularized) as well as for random forests. The stability with regard to removal of features with high discriminating power is finally equal to the classifiers’ performance within CSE-CIC-IDS2018. The results show that the property can thus stay intact. Fast convergence with subsequent invariability to training volume which also was a property within IDS2018 can be maintained as well. Any combination of models trained on either of the DDoS subsets of IDS2018, combined with either one of the NetBIOS DDoS subsets of DDoS2019 achieve these results, making them the most robust so far. The best classification scores achieved by pretrained models bar none are achieved by the randomized decision trees, a method that had faltered until now. With minmax-scaled features, it manages to reach almost perfect classification scores (recall: 99.88% with 99.7% precision). Unfortunately, these models do not have stable profiles with regard to training volume or feature reduction, making them less reliable overall.

- NTP

The monlist command in implementations of the network time protocol (NTP) used for clock synchronization between hosts is perhaps the best-known amplification DDoS attack. Like the others, it works over UDP, but unlike the others NTP reflection attacks have an amplification factor of more than 550. The command is a diagnostic tool that gives a list of 600 clients that recently made use of the NTP server. Subset 4 of CICDDoS2019 contains the NTP amplification attack samples. By now, the pretrained models that have shown good stable performance in the previous sections only have to continue exhibiting this behavior to solidify their position. This does appear to be the case with all tree-based methods scoring high (perfect recall with 60+% precision). Stability is strong for methods such as the bagging classifier, random forests and the gradient-boosted trees. There are some discrepancies between the models pretrained on DDoS subset 1 of IDS2018 compared to the models pretrained on subset 2 of the same dataset. That does weaken the results somewhat.

- SNMP

**Figure 13:**
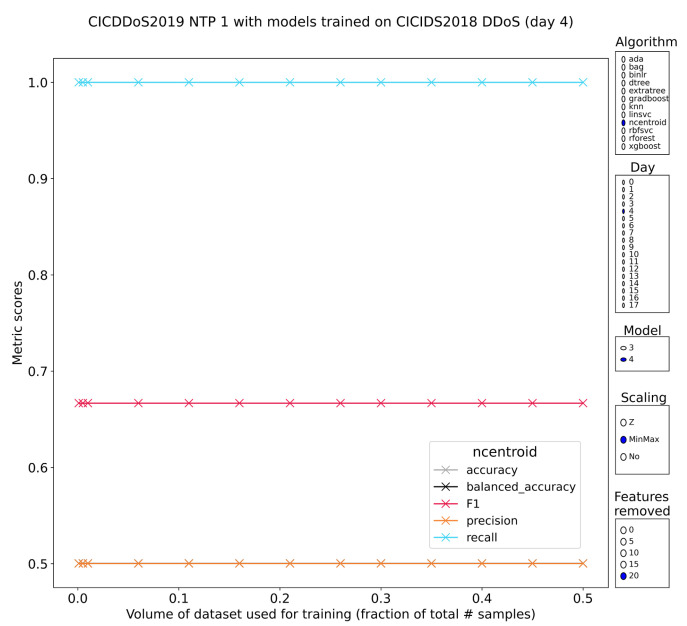
The constant behavior of nearest-centroid models when blindly evaluating DDoS samples from CIC-DDoS-2019. The method maintains perfect recall, but precision is heavily affected, falling back to a mere 50%.

SNMP amplification abuses the GET BULK request type of the protocol which bundles smaller amounts of information that would otherwise have to be retrieved through several GET NEXT calls. Like the other attacks it is wrongly configured SNMP servers that respond to illegitimate use of a legitimate feature that causes the problem. Classification of this subset by the models pretrained on IDS2018 DDoS loses the roughly equal performance by models trained on either of the subsets. Several methods also perform worse in absolute terms on this specific attack that had been strong performers for the other variants (adaboost, extratree and xgboost spike high but have no consistency anymore). Good classifiers maintain large stable sections with perfect recall and 60–70% precision. The single constant so far have been nearest-centroid models, which score with identical performance numbers on all subsets (perfect recall and ~50% precision, Figure 13).

- SSDP

**Figure 14:**
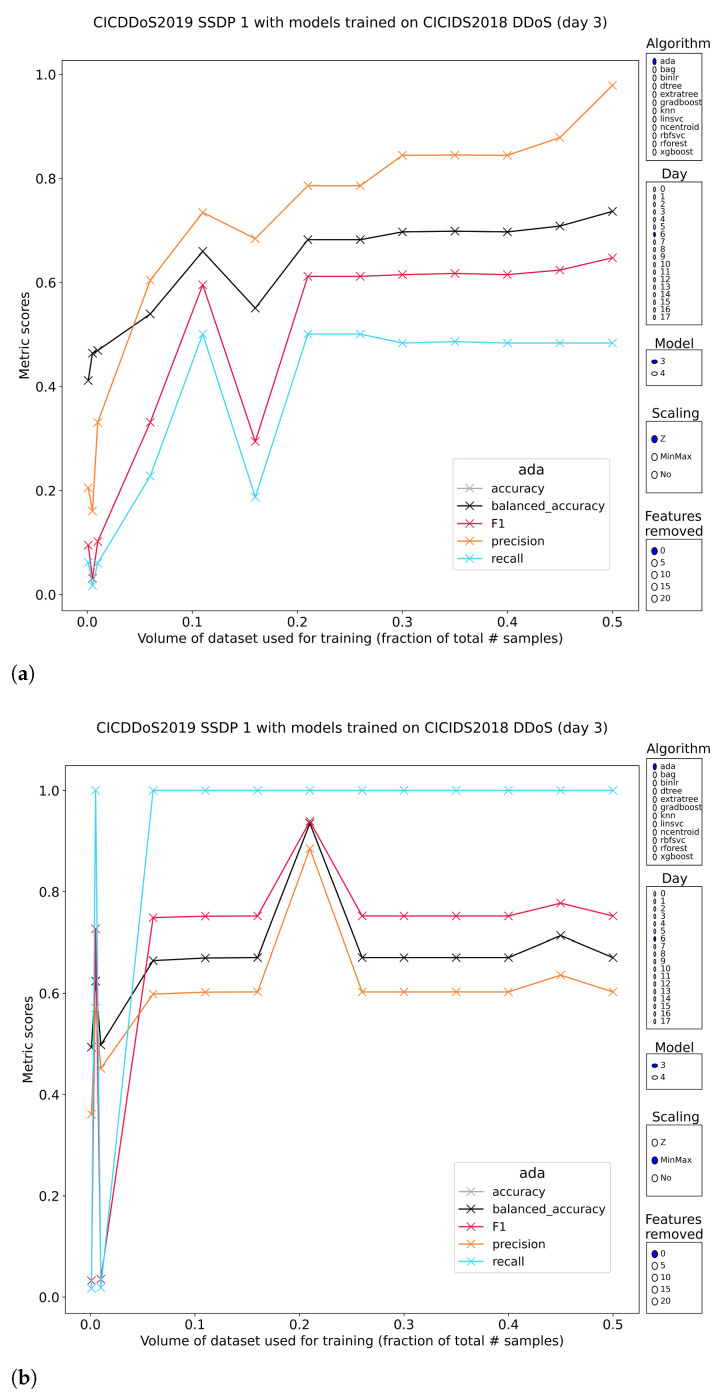
Feature scaling prior to training has a significant impact on performance in the interdataset case (**a**,**b**). The same methods did not show this weakness when just evaluating intradataset.

Simple Service Discovery Protocol (SSDP) is a part of the Universal Plug and Play (UPnP) protocols, allowing seamless discovery and access of services on a network by interested clients. The devices themselves can announce their presence and capabilities on the network with passive listeners, but an active version exists where a newly connected device can query the network for services to which it would like to connect. An M-SEARCH query looking to obtain responses from all service endpoints can easily reach more than 20× bandwidth amplification. SSDP amplification attacks were recognized fairly well both by tree-based models trained on standardized features as well as minmax-scaled features. This is rare, because most tree-based models built on normalized features have terrible performance across the board. It should be noted that the recall of models on standardized features is about half that of models trained on minmax-scaled features (Figure 14). Performance on the whole is consistent with the previous attack types. Still, even the highest performing pretrained models do not have equal performance if their data was either the first or second subset of DDoS traffic of CSE-CIC-IDS2018. These concerns keep on pushing current viability down, while at the same time opening new lines of research into robust tree-based models for intrusion detection.

- TFTP

**Figure 15:**
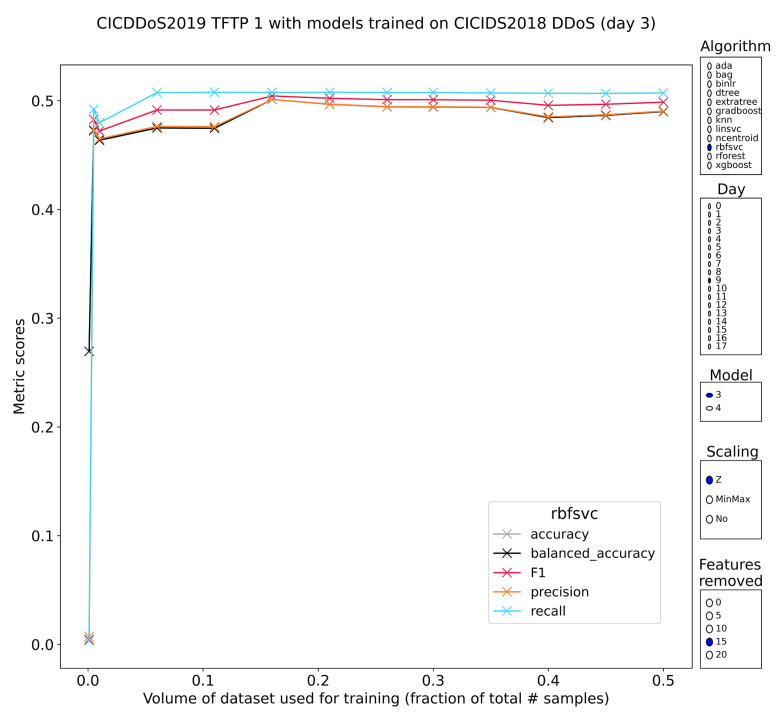
Even though performance is not high in absolute terms, the regression models and SVMs can exhibit clustered performance metrics. If these can be lifted to a higher plateau, then the methods could become viable.

The trivial file transfer protocol (TFTP) is a lightweight version of FTP that works over UDP. It has primarily seen adoption in network-booted environments. It was never intended to be exposed to the Internet, but as with all of these reflection attacks, misconfigured TFTP and firewalls allow misuse of the protocol. Recognition by the pretrained models follows the pattern of SSDP with mostly tree-based models that perform well and with some stability even retaining lots of performance if the features were normalized prior to training. This indicates that it is no fluke and while models built on minmax-scaled features still outperform, it might be possible to abstract over this choice altogether with the right training scheme. One other algorithm that has the interesting property of stable metrics that are tightly clustered and stable with regard to training volume and feature reduction is the RBF-kernel SVM. All metrics are densely packed around 55%, which is not high enough to be immediately usable, but the tightly clustered metrics are interesting (Figure 15). They have also been observed for the logistic regression and linear SVM (albeit at much lower values ~10–20). Perhaps these models could be improved while retaining the metric clusters and stability.

- Portmap

The portmapper service (also known as rpcbind) provides a linking of program number, program version and listening port. The client can ask for this information and then pick a preferred level to interact with the available service(s) through remote procedure calls (RPC). This access should be restricted to authorized users, but because there is no authentication/authorization by default, attackers can simply ask for a listing of the entire table and send it to a spoofed victim because UDP is available as the transport layer. Results on this class are almost equal to those on the previous classes. Decent patches of performance exist, but the variability is large and no overall recommendations can be made that will guarantee stable, high performance. There is almost no agreement between the pretrained models on subset 1 of DDoS from IDS2018 versus those pretrained on subset 2. This issue has been intermittently showed up for the other attack classes as well. Good performance in numbers for this class ranges from perfect recall paired with 60% precision, which can be fairly stable, to models with recall–precision pairs above 99.5% without stability.

#### 3.5.2. Exploitation-Based DDoS

The attacks described in the previous Section 3.5.1 all rely on exploiting available commands in protocols that through misconfigured daemons respond to illegitimate requests. The power of those attacks is in the amplification factor, which enables an attacker to use fewer resources but still reflect massive amounts of traffic to a spoofed target. The three attacks described in this section are less sophisticated but require more resources on the attacker’s side (e.g., a larger botnet). These attacks can use IP spoofing, but they stay effective without it.

- UDP-flood

UDP-floods simply overwhelm a server by sending packets to random UDP ports, not even interested in a particular service. The OS on the receiving end has to process the packet and potentially send an ICMP packet back. If there is a firewall between the target and the attacker that will take the first hit, but it too has to handle the traffic somehow. Even if it manages to ignore the majority of malicious packets, the link itself can get filled up with the packets. Despite their simplicity, these attacks can have the same practical outcome as the reflection-based DDoS attacks. Basic UDP floods are well-recognized, with some degree of consistency between the models and relative stability. This applies most to the tree-based meta-estimators. Raw performance numbers are perfect recall with 60–92% precision. Minmax scaling had to be applied to the inputs before training for these results. Models trained with normalized features can still be decent, up to perfect recall and 89% precision, but hampered by stability issues. These conclusions apply to both subsets with UDP-flood traffic (7 & 16).

- SYN-flood

**Figure 16:**
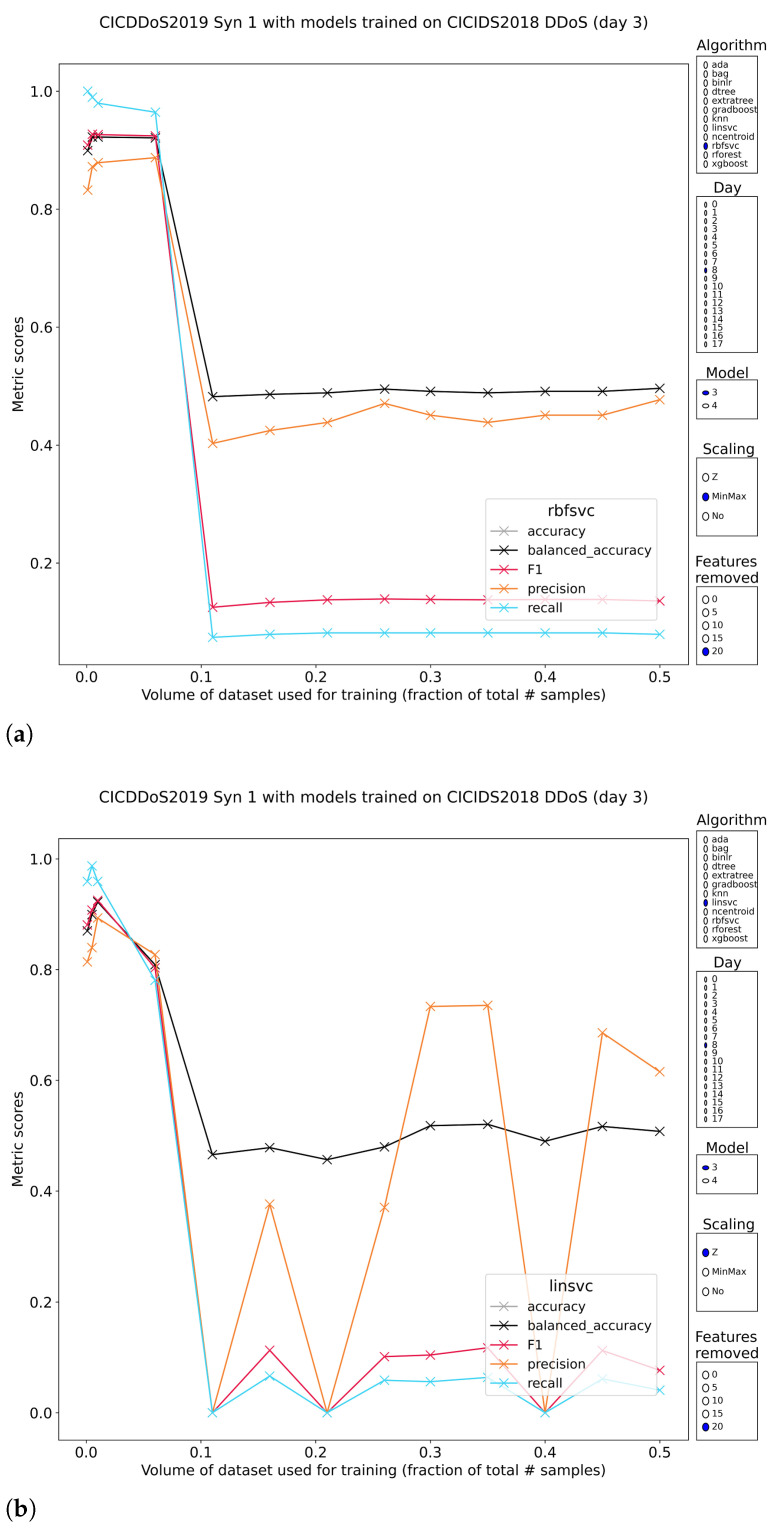
Another instance where, contrary to standard assumptions, increasing the training volume has a clear negative effect on generalized performance (**a**,**b**).

SYN floods work by an attacker creating lots of half-open TCP connections (client SYN, server SYN/ACK, client does not send ACK) but never actually using them. Because the number of connections always has an upper bound, it is only a matter of resources to tie up all available connections. The result pattern for SYN-flood attacks is strange. On the one hand, the models pretrained on DDoS subset 1 of CSE-CIC-IDS2018 do not perform well at all, except for some cases where high classification metrics are posted, but only at minute training volumes (<1%). Models trained on DDoS subset 2 of IDS2018 score much better, but even the tree-based models no longer hold perfectly stable recall. The observation that some of the best models were trained on little data, flows to lesser or greater extent through all attack types. It is possible that for clear network attacks, limited exposure is enough to achieve robustness, rather than the typical assumption that more data is better (Figure 16).

- UDP-lag

**Figure 17:**
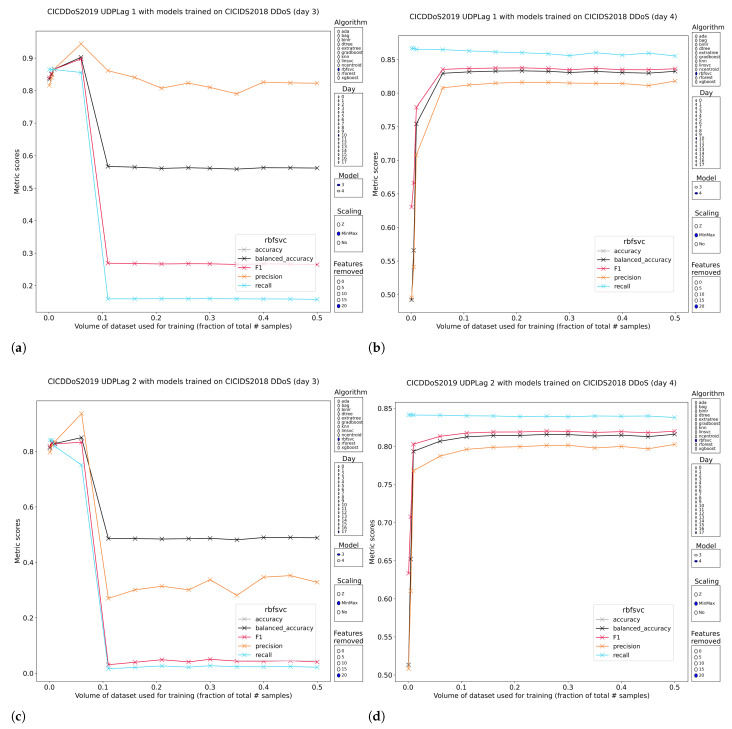
RBF-SVMs pretrained on the available DDoS subsets in CSE-CIC-IDS2018 have significantly different, but self-consistent general performance profiles on the UDP-lag attack subsets of CIC-DDoS2019.

The UDP-lag attack is a niche network attack whereby a client abuses an existing connection between itself and the server by introducing lag at the client side. Typically, clients do not immediately disconnect from the service as that would hurt the user experience but instead can continue local interaction with the service and the network will catch up when the link improves. This may create advantages for the client because the server is not in control and might have to deal with a state generated on the client side that would have been impossible if the connection had stayed intact. This attack is classified under DDoS because one way of introducing it is by DDoSing your own machine. The clearest case of requiring models to train on a limited amount of samples to have a good general representation is this DDoS subclass and the nearest neighbors algorithm. Even though it is so simple, F1-scores above 90% happen with a large degree of consistency, not just with regard to feature removal but also between the two sets of pretrained models and the two sets of UDP-lag traffic of CIC-DDoS2019. The only caveat is a serious performance regression when increasing the training volume (most visible with standardization). These performance regressions have been observed for the logistic regressions and SVMs for this and other attack types of this data set, but the slope of the decline is much less steep, because these models did not achieve good metrics to begin with. One final illustration that summarizes many of the observations in all the previous subsections is shown in quadrant Figure 17. The four images show how the RBF-kernel SVM’s performance can differ. The pair of pretrained models have perfect consistency on both subsets of UDP-lag traffic, but they are almost fully negatively correlated with each other.

#### 3.5.3. Intermediate Conclusion

When tasking CIC-IDS2018’s pretrained DDoS models with classifying CIC-DDoS2019, the interdataset generalization of these models can be summed up as follows. Classification results can be near-perfect and highly volatile (for tree-based learners) or significantly less accurate, but more often stable for SVMs and simple distance-based learners. Consistency when classifying is possible, but it is not the norm. Nevertheless, the existence of consistent sets of models indicates that it is possible to train them. Further investigation is required to compare this sparse set of stable, well-generalizing models to their failing counterparts. As a network-centric attack class, models with pockets of good generalization were expected and confirmed, but they exhibit the same flaws observed from generalization on the other datasets. The inconsistent loss of key intradataset generalization properties such as high discriminating power, stability with regard to data reduction and invariability to scaling seriously hamstrings dependable, effective use of these methods.

## 4. Discussion

The intermediate conclusions (Section 3.3.7 and Section 3.5.3) interspersed in the result Section 3 are meant to maintain oversight of the patterns observed in the result collection. However, in order to provide an even more succinct overview, this discussion is structured around Table 7, Table 8 and Table 9. Every table shows the baseline performance (B) of the top 3 models, selected based on an equally weighted combination of balanced accuracy, f1-score and training volume (lower is better). The performance metrics of the top 3 interdataset generalizing models on the corresponding attack class follow immediately. The classification metrics are percentages with a maximum of 100.

The tables show the numeric confirmation of the statement from the intermediate conclusions that it is possible to train models that generalize well to novel samples, generated by unseen attacks from the same class, executed in a different network environment. Structuring the entire result section around these results would have been misleading because the top 3 models obscure the loss of stability and invariance to data reduction and feature scaling.

Table 7 summarizes the generalized results from training on CSE-CIC-IDS2018 to classifying CIC-IDS2017. Generalization on the brute force attack category shows the smallest deviation between baseline and generalized performance (<1% loss in balanced accuracy). Losses of 10–15% in class-balanced accuracy are the norm. This loss still obscures information. The distinction in interdataset generalization performance is more noticeable when looking only at the classification of malicious samples. The F1-score typically drops 5–15 points for the network-centric attack classes (brute force, DoS, DDoS), but the other classes show drops of at least 20 points (botnet). Web attacks show the steepest overall loss of performance. F1-scores are reduced from north of 90% to anywhere between 10 and 70% and these are the performance numbers of the best-generalizing models. Precise recognition of infiltration attacks drops close to 0%, although it should be noted that the baseline performance on this class was the weakest to begin with. Recognition of botnet traffic is affected mostly on the recall front, which suggests that the best models have learned precise traffic profiles for the specific botnets. For this attack class, it may even be preferable to favor precision over recall to correctly identify infected machines.

Table 8 is a lot shorter, because it summarizes general performance of application layer DoS models, trained on CSE-CIC-IDS2018 to CIC-DoS2017. The conclusion in the result section (Section 3.4) for this target dataset already stated the underwhelming performance and this is reflected in the top 3 generalizing models. Recall consistently outstrips precision, but both are lackluster overall (never exceeding 80%). The mixture of a much larger variety of DoS attacks and attack-free traces from the older ISCXIDS2012 instead of the network setup for 2017’s datasets are the most likely culprits for the performance regression. Further investigation is required to parse the weights of each factor individually.

Finally, the generalized results of 2018’s DDoS models to every specific attack represented in CIC-DDoS2019 are listed in Table 9. Baseline performance in the DDoS category is close to perfect and this largely carries over to generalized performance. Recall is least affected, with drops nearly always capped at 5 points. Precision varies slightly more, with maximal drops around 15 points. An interesting phenomenon in the DDoS results is the consistency of certain models. The randomized decision trees classifier (extratree), with minmax scaled features, 10/15 top features removed and access to no more than 11% of the data to train and validate, appears frequently. Its generalization performance is thus quite stable across the novel DDoS attacks.

Generalizability will need improvement to start adopting learning IDS models. The major issue to resolve is consistency. In our opinion, four options are available. On the pure data science track, it is possible that models trained on an intelligent selection of features might suffer less from instability. Second, models with a higher capacity might achieve better abstracted versions of the attack classes. A third solution could lie in the expansion of the quality and variety of academic data available for model building. By doing so the inconsistency issue may be overcome because the models are trained on much more comprehensive samples of the attack classes. A fourth option would be to compare the sets of best-in-class general models for each attack class and compare their internals to those of the unsuccessful models trained for the same attack class. This would provide more insight into the factors that drive general performance. The future work and hypotheses Section 5.1 provides more details about these potential solutions.

## 5. Conclusions and Future Work

This work exposes supervised ML intrusion detection models with excellent recognition on CSE-CIC-IDS2018 to the novel samples of CIC-IDS2017, CIC-DoS2017 and CIC-DDoS2019. Those datasets share the included attack types but have been generated with mostly different individual attacks and in different network environments. Based on the results obtained by these models within CSE-CIC-IDS2018, it was assumed that the pretrained models would generalize well and successfully classify the other datasets’ samples. This assumption has been standard but untested in ML-based intrusion detection research, in part because of the low availability of compatible, labeled datasets. As a consequence, new proposed IDS systems are recognized as an improvement in the state-of-the-art if they succeed at reaching higher classification scores, with training, validation and testing limited to samples of one or two datasets without crossover testing. This work shows experimentally that the assumption that good intradataset generalization will yield good interdataset generalization (and further implied extradataset generalization) is faulty.

Models for attacks that rely heavily on network interaction tend to suffer the smallest performance regression in interdataset testing. Recognition of the malicious samples for the brute force, DoS and DDoS attack classes decreases by 5–15 points (from near-perfect classification F1>99% to 85%<F1<95%). Performance degradation in terms of balanced accuracy is typically lower, meaning that the loss of recognition impacts the malicious classes more than it does the benign class. Even for the network-centered classes, this conclusion is an overgeneralization because specific performance loss can be lower (e.g., for many attacks in CIC-DDoS2019) or higher (e.g., for CIC-DoS2017).

General performance in recognizing attacks that use the network for transport but are otherwise targeted more toward the disruption of the host can easily be summarized. Even for the highest-achieving models in every class, interdataset performance is poor to extremely poor. Recognition of botnet traffic drops in terms of recall from 99+% to 64% but keeps precision intact. Recognition of Web attacks loses a couple of points in recall, but more than 50 points in precision (~>95% to <30%), rendering the models useless. Interdataset recognition of infiltration traffic is nonexistent as generalized precision never exceeds 1%. The full tables with the performance numbers of the three best generalizing models for each attack class and each dataset are part of the discussion (Section 4).

Whereas data reduction (both features and samples) and feature scaling had little to no impact on the excellent intradataset classification ( [15]), in interdataset classification, these benefits are gone.

In short, it is impossible to tell in advance if a model will have a good general representation of the attack class. It is possible, but not the norm and metrics from the standard intradataset generalization overestimate real classification ability. Unless a solution is found that guarantees a model’s general performance, having to train and test many models and cherry-picking those with good intra- and interdataset performance, is a costly endeavor and a procedure that would still only be viable for the network-centered attack classes.

Ultimately, this work urges researchers to consider adopting a more rigorous validation approach that includes testing with unseen samples by default. Novel methods should be recognized as improvements to the state-of-the-art primarily by how well they continue to function in the most realistic test scenario available.

### 5.1. Future Work and Hypotheses

Several routes remain open for further investigation. First, the models in this work were pretrained as part of an earlier publication which investigated how far data could be reduced/damaged prior to training and still yield models with high discriminating power [15]. The poor interdataset results of these models may be due to them having had suboptimal access to data. Future work will train new models with only the highest quality features and even more strict control to prevent overfitting. If these maximally data-efficient models have stable, strong baseline performance and stable, strong interdataset performance, then the concerns raised in this article can be attenuated. If not, then the findings in this article will be reinforced.

A second route for future investigation is the inclusion of newer ML-based intrusion detection models. Many publications have adopted techniques from the deep learning research field and applied them to the IDS pattern recognition problem [52,53,54,55]. None of these articles however include a section on generalized performance. It is possible that these advanced models are able to capture the problem in a more robust way and, in doing so, eliminate the current gap. This hypothesis should be tested early to avoid building castles in the sky.

If neither classical models trained with optimal features or deep neural networks provide an answer for the generalization problem, then solutions three to five should be considered. Solution three consists of setting up new data generation experiments for intrusion detection. The emphasis should be on generation of samples from a large variety of modern attacks in variable, but controlled mirrors of realistic network environments. Variety and interoperability should be the main goals. In this regard, the LUFlow project [56] is interesting because it has captured compatible data between 2020 and 2022. This dataset directly enables work on interdataset generalization and concept drift across time.

Solution four includes comparing the rare instances of sets of models of the same algorithm that generalize well to their poorly generalizing counterparts. The focus should be on examining what makes these models different and if they can be adapted or trained in such a way that they are more likely to exhibit the desired behavior.

Solution five tailors IDS to a specific protocol. Rather than trying to build models that can work to detect misuse of any service on the network, specialized models could integrate knowledge of how specific protocols should operate. Within wireless intrusion detection, this approach has shown promising results [57].

## Figures and Tables

**Table 1:** CIC-IDS2017 Class Label Distribution.

Dataset	Subset Nr.	Attack Type	#Benign	#Malicious
	0	FTP-SSH brute force	432074	13835
	1	HTTP-DoS/DDoS	440031	252672
	2	Web attacks	168186	2180
CIC-IDS2017	3	Infiltration	288566	36
	4	Botnet	189067	1966
	5	DDoS	128027	97718
	6	Port scan	158930	127537

**Table 2:** CIC-DoS2017 Class Label Distribution.

Dataset	Subset Nr.	Attack Type	#Benign	#Malicious
CIC-DoS-2017	0	Combined	248896	42706

**Table 3:** CSE-CIC-IDS2018 Class Label Distribution.

Dataset	Subset Nr.	Attack Type	#Benign	#Malicious
	0	FTP-SSH brute force	667626	380949
	1	HTTP-DoS	996077	52498
	2	HTTP-DoS	446772	601802
	3	DDoS	576191	472384
CSE-CIC-IDS2018	4	DDoS	687742	360833
	5	Web attacks	1048213	362
	6	Web attacks	1048009	566
	7	Infiltration	544200	68871
	8	Infiltration	238037	93063
	9	Botnet	762384	286191

**Table 4:** CIC-DDoS-2019 Class Label Distribution.

Dataset	Subset Nr.	Attack Type	#Benign	#Malicious
	0	DNS	3403	3403
	1, 11	LDAP	1613, 5125	1613, 5125
	2, 12	MSSQL	2007, 2795	2007, 2795
	3, 13	NetBIOS	1708, 1322	1708, 1322
	4	NTP	14366	14366
CIC-DDoS-2019	5	SNMP	1508	1508
	6	SSDP	763	763
	7, 16	UDP	2158, 3135	2158, 3135
	8, 15	SYN	393, 35791	393, 35791
	9	TFTP	25248	25248
	10, 17	UDPLag	3706, 4069	3706, 4069
	14	Portmap	4735	4735

**Table 5:** Most discriminative features of CSE-CIC-IDS2018.

Dataset	Most Discriminative	
CSE-CIC-IDS2018	1–5	Timestamp, Init Win bytes forward, Destination Port, Flow IAT Min, Fwd Packets/s
	5–10	Fwd Packet Length Std, Avg Fwd Segment Size, Flow Duration, Fwd IAT Min, ECE Flag Count
	10–15	Fwd IAT Mean, Init Win bytes backward, Bwd Packets/s, Idle Max, Fwd IAT Std
	15–20	FIN Flag Count, Fwd Header Length, SYN Flag Count, Fwd Packet Length Max, Flow Packets

**Table 6:** Mapping of the subsets of CSE-CIC-IDS2018 to their counterpart in CIC-IDS2017.

Attack Class	2018	Tools	2017	Tools
Brute force	0	Patator.py (FTP/SSH)	0	Same attack tool
DoS layer-7	1	Slowloris Slowhttptest Hulk Goldeneye	1	Same attack tools
Heartbleed	2	Heartleech	1	Heartleech
DDoS	3	Low Orbit Ion Cannon (LOIC) (HTTP)	5	LOIC HTTP
DDoS	4	LOIC-UDP, High Orbit Ion Cannon (HOIC)	5	LOIC HTTP
Web attacks	5	Selenium (XSS, bruteforce), SQLi vs. DVWA	2	Same attack tools
Web attacks	6	Selenium (XSS, bruteforce), SQLi vs. DVWA	2	Same attack tools
Infiltration	7	Nmap, Dropbox download	3	Metasploit, Dropbox download
Infiltration	8	Nmap, Dropbox download	3	Metasploit, Dropbox download
Botnet	9	Zeus, ARES	4	ARES
Port scan	-	-	6	Various Nmap commands

**Table 7:** Classification metrics for the best 3 models per attack class, both for baseline (B) and generalized (G) classification, with mention of the preprocessing parameters.

B/G	Class	M	Algorithm	Balanced Acc.	F1	Precision	Recall	Scaling	Reduction	% Train
B	18-0.Bruteforce	18-0	rforest	100.00	100.00	100.00	100.00	MinMax	0	0.1
			gradboost	100.00	99.99	99.99	100.00	No	0	0.1
			bag	99.99	99.98	99.95	100.00	No	0	0.1
G	17-0.Bruteforce	18-0	gradboost	99.81	94.61	89.79	99.98	No	0	0.1
			dtree	99.81	94.59	89.75	99.98	No	0	0.5
			gradboost	99.76	94.57	89.79	99.89	No	0	1.0
B	18-1.L7-DoS	18-1	ada	100.00	99.93	99.87	100.00	MinMax	0	0.5
			dtree	100.00	99.91	99.82	100.00	No	0	0.5
			dtree	99.87	99.61	99.47	99.76	Z	0	0.1
B	18-2.L7-DoS	18-2	xgboost	99.98	99.99	99.97	100.00	MinMax	0	0.1
			rforest	99.98	99.99	99.98	100.00	MinMax	0	0.1
			dtree	99.98	99.99	99.97	100.00	MinMax	0	0.1
G	17-1.L7-DoS	18-1	ada	93.53	92.54	96.43	88.94	No	0	11.0
			ada	92.64	91.11	93.57	88.78	MinMax	0	11.0
			ada	87.84	83.89	79.31	89.02	No	0	1.0
G	17-1.L7-DoS	18-2	extratree	88.22	84.26	79.19	90.04	No	5	6.0
			extratree	87.51	82.80	74.03	93.94	No	0	6.0
			extratree	88.46	84.58	79.71	90.09	No	5	11.0
B	18-3.DDoS	18-3	gradboost	99.81	99.83	99.87	99.79	MinMax	0	0.1
			xgboost	99.80	99.83	99.72	99.94	MinMax	0	0.1
			knn	99.80	99.83	99.72	99.94	MinMax	0	0.1
B	18-4.DDoS	18-4	extratree	99.99	100.00	99.99	100.00	No	20	0.1
			ada	99.98	99.99	99.97	100.00	MinMax	5	0.1
			extratree	99.98	99.99	99.98	100.00	Z	5	0.1
G	17-5.DDoS	18-3	knn	91.29	90.70	98.99	83.70	Z	15	0.1
			knn	88.88	87.70	99.18	78.61	Z	5	0.1
			binlr	86.58	89.05	86.73	91.50	Z	15	0.1
G	17-5.DDoS	18-4	linsvc	89.42	90.81	90.91	90.71	MinMax	15	1.0
			xgboost	86.68	88.79	87.66	89.95	MinMax	15	6.0
			linsvc	81.02	86.82	78.29	97.42	MinMax	15	0.5
B	18-5.Web	18-5	xgboost	96.96	96.87	100.00	93.92	Z	0	6.0
			xgboost	98.90	98.88	100.00	97.79	MinMax	0	11.0
			dtree	97.93	91.80	88.07	95.86	Z	0	6.0
B	18-6.Web	18-6	xgboost	95.49	95.19	99.81	90.99	Z	0	11.0
			xgboost	96.82	96.28	99.07	93.64	No	0	16.0
			xgboost	96.29	96.06	99.81	92.58	MinMax	0	16.0
G	17-2.Web	18-5	ncentroid	90.34	42.42	28.44	83.39	MinMax	15	35.0
			ncentroid	84.62	9.51	5.01	91.79	No	15	1.0
			ncentroid	84.62	9.51	5.01	91.79	No	20	1.0
G	17-2.Web	18-6	xgboost	91.13	71.47	62.81	82.89	MinMax	0	21.0
			ada	91.24	30.87	18.74	87.39	MinMax	15	0.5
			ada	94.54	46.19	30.87	91.74	MinMax	0	35.0
B	18-7.Infil	18-7	gradboost	74.88	37.13	23.88	83.39	Z	0	0.1
			ada	69.73	46.62	47.32	45.93	No	0	6.0
			ada	69.47	46.50	47.94	45.14	Z	0	6.0
B	18-8.Infil	18-8	dtree	93.31	86.96	79.06	96.63	No	0	1.0
			dtree	92.33	86.35	79.95	93.87	MinMax	0	1.0
			dtree	92.08	85.80	78.96	93.95	MinMax	0	0.5
G	17-3.Infil	18-7	ada	85.19	0.40	0.20	75.00	Z	15	0.1
			bag	84.06	1.30	0.65	69.44	MinMax	5	0.1
			gradboost	83.43	0.18	0.09	77.78	MinMax	20	1.0
G	17-3.Infil	18-8	dtree	88.84	0.69	0.35	80.56	Z	15	1.0
			gradboost	83.71	0.13	0.07	83.33	MinMax	10	0.5
			gradboost	81.07	0.12	0.06	77.78	Z	20	0.1
B	18-9.Botnet	18-9	rforest	99.91	99.88	99.89	99.87	Z	0	0.1
			extratree	99.88	99.86	99.95	99.77	Z	0	0.1
			extratree	99.89	99.81	99.77	99.86	No	0	0.1
G	17-4.Botnet	18-9	gradboost	82.07	78.13	99.92	64.14	No	0	0.5
			bag	82.07	78.10	99.84	64.14	No	0	1.0
			xgboost	82.06	77.53	97.98	64.14	No	0	0.5

**Table 8:** CIC-DoS2017 data classification metrics by DoS models pretrained on CSE-CIC-IDS2018 for the best 3 models per attack class, both for baseline (B) and generalized (G) classification, with mention of the preprocessing parameters.

B/G	Class	M	Algorithm	Balanced Acc.	F1	Precision	Recall	Scaling	Reduction	% Train
B	18-1.L7-DoS	18-1	ada	100.00	99.93	99.87	100.00	MinMax	0	0.5
			dtree	100.00	99.91	99.82	100.00	No	0	0.5
			dtree	99.87	99.61	99.47	99.76	Z	0	0.1
B	18-2.L7-DoS	18-2	xgboost	99.98	99.99	99.97	100.00	MinMax	0	0.1
			rforest	99.98	99.99	99.98	100.00	MinMax	0	0.1
			dtree	99.98	99.99	99.97	100.00	MinMax	0	0.1
G	0.L7-DoS	18-1	ncentroid	79.56	60.07	52.68	69.89	No	10	0.1
			ncentroid	78.74	59.31	52.58	68.01	No	0	0.5
			ncentroid	78.65	58.89	51.83	68.17	No	0	1.0
G	0.L7-DoS	18-2	bag	75.56	48.15	35.68	74.02	MinMax	5	0.1
			ada	74.64	44.63	30.99	79.74	Z	20	0.5
			bag	71.61	45.84	36.49	61.63	MinMax	10	0.5

**Table 9:** Classification metrics for the best 3 models per attack class, both for baseline (B) and generalized (G) classification, with mention of the preprocessing parameters.

B/G	Class	M	Algorithm	Balanced Acc.	F1	Precision	Recall	Scaling	Reduction	% Train
B	18-3.DDoS	18-3	gradboost	99.81	99.83	99.87	99.79	MinMax	0	0.1
			xgboost	99.80	99.83	99.72	99.94	MinMax	0	0.1
			knn	99.80	99.83	99.72	99.94	MinMax	0	0.1
B	18-4.DDoS	18-4	extratree	99.99	100.00	99.99	100.00	No	20	0.1
			ada	99.98	99.99	99.97	100.00	MinMax	5	0.1
			extratree	99.98	99.99	99.98	100.00	Z	5	0.1
G	19-0.DNS	18-4	bag	91.28	91.96	85.35	99.68	Z	15	0.1
		18-4	xgboost	88.45	89.61	81.40	99.68	Z	15	0.5
		18-4	xgboost	96.15	96.29	92.92	99.91	MinMax	15	21.0
G	19-1.LDAP	18-3	xgboost	98.98	98.98	98.35	99.63	MinMax	10	1.0
		18-3	ada	98.11	98.13	96.97	99.32	Z	0	6.0
		18-3	dtree	97.98	98.01	96.74	99.32	Z	20	6.0
G	19-11.LDAP	18-3	ada	87.98	88.27	86.16	90.50	MinMax	5	1.0
		18-3	rforest	87.54	87.90	85.44	90.50	MinMax	0	0.5
		18-3	dtree	87.69	88.02	85.68	90.50	MinMax	20	1.0
G	19-2.MSSQL	18-3	ada	93.64	94.02	88.72	100.00	MinMax	5	0.1
		18-3	rforest	93.69	94.07	88.80	100.00	MinMax	20	0.5
		18-3	bag	93.64	94.02	88.72	100.00	MinMax	5	1.0
G	19-12.MSSQL	18-3	rforest	91.75	92.38	85.86	99.96	MinMax	0	0.5
		18-3	ada	91.23	91.94	85.08	100.00	MinMax	5	0.1
		18-3	ada	91.32	92.01	85.21	100.00	MinMax	5	1.0
G	19-3.NetBIOS	18-3	extratree	99.77	99.77	99.77	99.77	MinMax	10	6.0
		18-3	xgboost	97.10	97.05	98.91	95.25	MinMax	10	1.0
		18-4	extratree	99.30	99.29	99.70	98.89	MinMax	15	11.0
G	19-13.NetBIOS	18-3	xgboost	97.99	98.03	96.28	99.85	MinMax	10	1.0
		18-3	extratree	99.77	99.77	99.70	99.85	MinMax	10	6.0
		18-3	gradboost	99.36	99.36	98.88	99.85	Z	20	6.0
G	19-4.NTP	18-3	extratree	99.48	99.48	99.53	99.44	MinMax	10	6.0
		18-3	xgboost	95.30	95.12	98.92	91.60	MinMax	10	1.0
		18-4	extratree	99.54	99.54	99.65	99.43	MinMax	20	11.0
G	19-5.SNMP	18-3	xgboost	95.72	95.58	98.73	92.63	MinMax	10	1.0
		18-3	bag	94.86	95.11	90.67	100.00	MinMax	0	1.0
		18-3	bag	94.72	94.99	90.46	100.00	MinMax	20	1.0
G	19-6.SSDP	18-3	extratree	99.34	99.34	99.60	99.08	MinMax	10	6.0
		18-3	extratree	98.95	98.94	99.87	98.03	MinMax	20	11.0
		18-4	extratree	98.62	98.61	99.60	97.64	MinMax	15	11.0
G	19-7.UDP	18-3	extratree	99.75	99.75	99.63	99.86	MinMax	10	6.0
		18-4	extratree	99.65	99.65	99.58	99.72	MinMax	15	11.0
		18-3	gradboost	96.94	97.03	94.39	99.81	MinMax	15	6.0
G	19-16.UDP	18-3	extratree	99.47	99.47	99.43	99.52	MinMax	10	6.0
		18-4	extratree	99.20	99.20	99.05	99.36	MinMax	15	11.0
		18-3	gradboost	94.51	94.80	90.11	100.00	MinMax	15	6.0
G	19-8.SYN	18-4	knn	98.21	98.25	96.55	100.00	Z	20	0.5
		18-3	knn	96.68	96.66	97.41	95.92	Z	5	0.1
		18-3	rbfsvc	95.54	95.39	98.64	92.35	Z	20	0.5
G	19-15.SYN	18-4	knn	97.81	97.85	96.00	99.78	Z	20	0.5
		18-3	rbfsvc	94.63	94.84	91.25	98.71	MinMax	5	1.0
		18-3	rbfsvc	94.14	94.41	90.26	98.96	MinMax	15	1.0
G	19-9.TFTP	18-3	ada	92.50	92.97	87.49	99.18	MinMax	5	0.1
		18-3	rforest	92.67	93.12	87.75	99.18	MinMax	20	0.5
		18-3	rforest	92.53	92.99	87.53	99.18	MinMax	0	0.5
G	19-10.UDPLag	18-3	knn	91.81	91.30	97.37	85.94	Z	5	0.1
		18-3	ncentroid	91.73	91.27	96.62	86.48	Z	15	0.1
		18-3	ncentroid	91.57	91.11	96.27	86.48	Z	5	0.1
G	19-17.UDPLag	18-3	ncentroid	90.51	89.86	96.47	84.10	Z	15	0.5
		18-3	knn	89.75	88.89	97.01	82.03	Z	10	0.1
		18-3	ncentroid	89.13	88.56	93.52	84.10	Z	15	0.1
G	19-14.Portmap	18-3	gradboost	99.14	99.14	99.34	98.94	Z	20	6.0
		18-3	dtree	93.05	93.44	88.51	98.94	Z	20	1.0
		18-4	extratree	97.06	97.13	95.10	99.24	MinMax	10	11.0

## Data Availability

The datasets, both raw and cleaned up, are publicly available at https://gitlab.ilabt.imec.be/lpdhooge/ids-dataset-collection. The full source code of the analysis and the complete set of visualizations is available at https://gitlab.ilabt.imec.be/lpdhooge/reduced-unseen-testing.
